# Endothelial Damage in Acute Respiratory Distress Syndrome

**DOI:** 10.3390/ijms21228793

**Published:** 2020-11-20

**Authors:** Alice G. Vassiliou, Anastasia Kotanidou, Ioanna Dimopoulou, Stylianos E. Orfanos

**Affiliations:** 1 1st Department of Critical Care Medicine & Pulmonary Services, School of Medicine, National and Kapodistrian University of Athens, Evangelismos Hospital, 106 76 Athens, Greece; alvass75@gmail.com (A.G.V.); akotanid@med.uoa.gr (A.K.); idimo@otenet.gr (I.D.); 2 2nd Department of Critical Care, School of Medicine, National and Kapodistrian University of Athens, Attikon Hospital, 124 62 Athens, Greece

**Keywords:** ARDS, dysfunction, biomarkers, coagulation, inflammation

## Abstract

The pulmonary endothelium is a metabolically active continuous monolayer of squamous endothelial cells that internally lines blood vessels and mediates key processes involved in lung homoeostasis. Many of these processes are disrupted in acute respiratory distress syndrome (ARDS), which is marked among others by diffuse endothelial injury, intense activation of the coagulation system and increased capillary permeability. Most commonly occurring in the setting of sepsis, ARDS is a devastating illness, associated with increased morbidity and mortality and no effective pharmacological treatment. Endothelial cell damage has an important role in the pathogenesis of ARDS and several biomarkers of endothelial damage have been tested in determining prognosis. By further understanding the endothelial pathobiology, development of endothelial-specific therapeutics might arise. In this review, we will discuss the underlying pathology of endothelial dysfunction leading to ARDS and emerging therapies. Furthermore, we will present a brief overview demonstrating that endotheliopathy is an important feature of hospitalised patients with coronavirus disease-19 (COVID-19).

## 1. Introduction

Using the updated Berlin definition, acute respiratory distress syndrome (ARDS) is defined as a syndrome of acute onset, with bilateral diffuse infiltrates on chest radiography, and non-cardiogenic respiratory failure, leading to mild, moderate, or severe oxygenation impairment [1]. Pathophysiologically, it is characterized by damage to the capillary endothelium and alveolar epithelium, and fluid accumulation in the alveolar space, leading to alveolar oedema. These changes in the microvascular endothelial structure and function play a central role in the acute inflammatory response, in which the body tries to eliminate microbial invaders. To achieve this, the endothelium becomes leaky and inflamed, allowing innate immune cells and humoral effector molecules to cross the barrier to the site of infection [2]. When the phenomenon becomes overwhelming, it leads to ARDS, whose inciting events can be either direct (mainly pneumonia, aspiration of gastric contents) or indirect (mainly sepsis, multiple trauma) insults to the lung, with sepsis and pneumonia being the most common cause of ARDS in humans [3]. The incidence of ARDS varies widely, from 15 to 70 cases per 100,000 persons per year, representing approximately 5% of hospitalized, mechanically ventilated patients [4]. Understanding the molecular mechanisms of endothelial dysfunction has potential diagnostic, prognostic, and therapeutic implications for this fatal disease [5,6,7]. The old term acute lung injury (ALI) was used in clinical studies, along with the term ARDS, until the Berlin definition was released, and it is still being used in experimental models [3].

## 2. Pathogenesis

A single layer of endothelial cells (ECs) lines the entire vascular system, and this vascular endothelium forms the innermost layer of all blood vessels. In the past, the vascular endothelium was considered to be inert and nothing more than a nucleated layer. However, it is now clear that it actively participates in several key functions including angiogenesis, blood clotting, vasomotor tone, and inflammation [8]. Moreover, endothelial cells produce various cytokines and adhesion molecules [9].

As discussed in detail below, endothelial dysfunction is characterized by a change in EC functions. These include increased permeability leading to vascular leakage and oedema formation; disruption of the balance between vasodilators and vasoconstrictors; pro-inflammatory characteristics, including increased expression of adhesion molecules, receptors and signal transduction molecules, as well as release of reactive oxygen species; pro-coagulant and anti-fibrinolytic phenotype, miscommunication with adjacent vascular cell wall.

## 3. Pulmonary Endothelial Functions

**Figure 1:**
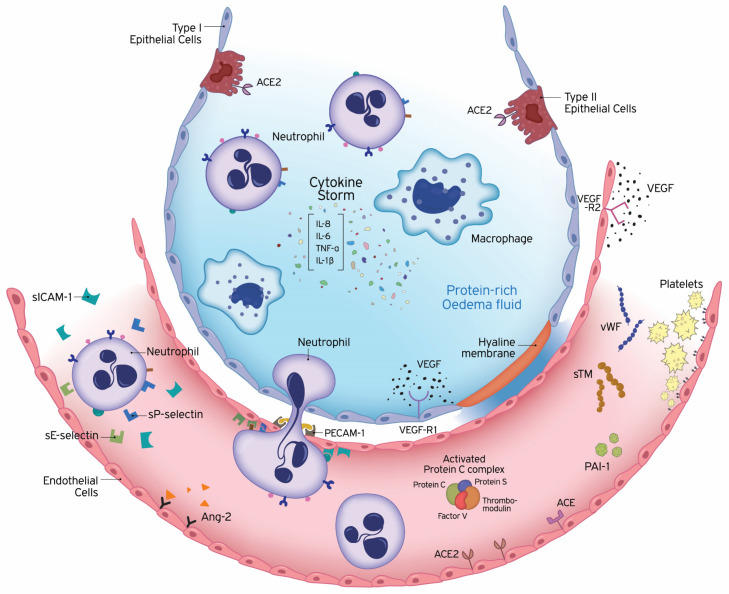
Underlying pathology of endothelial dysfunction leading to acute respiratory distress syndrome (ARDS). ARDS is characterized by damage to the capillary endothelium and alveolar epithelium. Disruption of the endothelial barrier results in the movement of fluid and macromolecules into the interstitial space and pulmonary air spaces causing pulmonary oedema. The formation of a hyaline membrane in alveolar walls allows exudation of neutrophils and protein-rich fluid into the alveolar space. Transport across the endothelium can occur either via the endothelial cell (transcellular) or between adjacent cells, through inter-endothelial junctions (IEJs) (paracellular). The changes in the microvascular endothelial structure and function play a central role in the acute inflammatory response, in which the body tries to eliminate microbial invaders. To achieve this, the endothelium becomes leaky and inflamed, allowing innate immune cells and humoral effector molecules to cross the barrier to the site of infection. This defence mechanism may become deleterious, under overwhelming pathological conditions, leading to ARDS. The cells of the innate immune system release large amounts of pro- and anti-inflammatory cytokines, such as IL-1β, IL-6, IL-8, and TNF-α. The high levels of circulating cytokines can potentiate organ damage by endothelial injury and other routes. Endothelial damage is associated with activation of neutrophils and expression of neutrophil and endothelial adhesion molecules. E-selectin and P-selectin are early mediators of the adhesion of activated neutrophils to endothelia in inflammatory states, prior to their firm adhesion and diapedesis at sites of tissue injury and inflammation. Intercellular adhesion molecule-1 (ICAM-1) controls the firm adhesion of neutrophils on the endothelium and facilitates their subsequent transendothelial migration via the platelet-endothelial cell adhesion molecule-1 (PECAM-1) to infection sites. In addition to inflammation, coagulation and fibrinolysis are also critical host responses to infection and injury involved in ARDS. Endothelial cells (ECs) coordinate this response by shifting from their normal anti-thrombotic, anti-inflammatory, and pro-fibrinolytic phenotype to an activated state of endothelial dysfunction. ECs actively regulate haemostasis by producing a variety of proteins, including pro-thrombotic substances (von Willebrand factor, P-selectin), molecules restricting coagulation (thrombomodulin) and fibrinolytic factors (plasminogen activators). Plasminogen activator inhibitor-1 (PAI-1) is the major inhibitor of fibrinolysis, whose upregulation leads to a shift from pro- to anti-fibrinolytic phenotypes. The protein C (PC) system provides important control of coagulation by virtue of the capacity of activated protein C (APC) to proteolytically inactivate the cofactors Va and VIIIa. The PC anticoagulant system also involves protein S, and the endothelial receptor thrombomodulin (TM). Conversion of protein C to the anti-coagulant APC is generated by TM-bound thrombin. The vascular endothelium has an important metabolic function with respect to vasoactive substances. Several vasoconstrictors and vasodilators are produced by the endothelium, such as endothelin-1, angiotensin-2, nitric oxide, and prostacyclin, which regulate vasomotor tone and the recruitment and activity of inflammatory cells and regulate thrombosis. In addition to breaking down bradykinin, ACE hydrolyses angiotensin I to angiotensin II and the balance between ACE and ACE2 has been suggested to be crucial for controlling angiotensin II levels. Vascular development strongly depends on the collaboration of growth factors. Vascular endothelial growth factor (VEGF) is a glycoprotein originally isolated as a permeability factor with unique specificity for vascular ECs. Angiopoietin-2 (Ang-2) disrupts the protective effects of Ang-1-Tie2 signalling, promoting vascular leakage. Ang-1, angiopoietin-1; Ang-2, angiopoietin-2; APC, activated protein C; IEJs, inter-endothelial junctions; IL, interleukin; PAI-1, plasminogen activator inhibitor 1; PECAM-1, platelet-endothelial cell adhesion molecule-1; sICAM-1, soluble intercellular adhesion molecule-1; sTM, soluble thrombomodulin; TNF-α, tumour necrosis factor-alpha; VEGF, vascular endothelial growth factor; VEGF-R, VEGF receptor; vWF, von Willebrand factor.

The vascular endothelium is a highly specialized metabolically active organ with many physiological, immunological, and synthesizing functions (Table 1). In this review, we will focus on changes occurring in some of these functions in ARDS (Figure 1). We will discuss these changes in both the clinical and preclinical context. Most animal models of ALI are based on clinical disorders that can lead to the development of ARDS in humans, such as sepsis, trauma, aspiration of gastric contents, and reperfusion of ischemic tissues. The animal models reproducing these risk factors and most suitable for the study of ARDS are related to ventilator-induced lung injury (VILI), lipopolysaccharide (LPS), live bacteria, hyperoxia, bleomycin (BLM), oleic acid, cecal ligation and puncture and acid aspiration [10].

### 3.1. Endothelium Barrier and Transport Functions

The endothelium, apart from being a semipermeable barrier separating blood from the surrounding tissues and, in the lungs, blood from the air, also regulates the transport of fluid and solutes between the blood and the interstitial space [8]. Disruption of the endothelial barrier results in the movement of fluid and macromolecules into the interstitial space and pulmonary air spaces causing pulmonary oedema. Transport across the endothelium can occur either via the endothelial cell (transcellular) or between adjacent cells, through inter-endothelial junctions (IEJs) (paracellular) [11,12]. IEJs are composed of tight junctions (TJs), adherens junctions (AJs), and gap junctions (GJs), which interact with integrin receptors to support EC adhesion to the underlying matrix [13]. TJs, formed by occludin, claudins, and junctional adhesion molecules (JAMs), act as a selective barrier to the entrance of molecules from the circulation; AJs, formed by vascular endothelial cadherin (VE-cadherin), mediate cell-to-cell contact and have a central role in barrier function, while GJs, which are formed by connexins, facilitate direct cell-to-cell transfer of signalling molecules, ions, and transmembrane potential [13,14]. Solutes and water can also cross the endothelial barrier via a transcellular pathway. Vesicles (or caveolae) have long been considered a pathway for the exchange of plasma proteins between the blood and interstitial compartment [15]. Although vascular permeability depends on both the tight junctions and caveolae, oedema develops mainly as a result of dysfunction of tight junctions [9]. Persistent opening of intercellular junctions leads to the formation of protein-rich oedema in the interstitial tissue, the main characteristic of tissue inflammation that may cause fatal diseases such as ARDS [16]. Thus, understanding the signalling pathways that prevent the disruption of endothelial barrier functions will be important for reversing ARDS and other diseases occurring as a consequence of such disruption. Indeed, a very recent study has shown that treatment with unfractionated heparin alleviated sepsis-induced lung injury in vivo by protecting TJs in lung microvascular endothelial cells (LMVECs) [17]. IEJs are also covered by a layer of fibrous matrix, the endothelial glycocalyx (EG). Dysfunction of the glycocalyx can also cause microvascular leakage, and evidence of EG shedding was discovered in ARDS established after flu syndrome [18]. Moreover, the carotenoid chemical compound, crocin, alleviated LPS-induced ARDS by means of protecting against glycocalyx damage [19].

### 3.2. Vascular Tone

The vascular endothelium has an important metabolic function with respect to vasoactive substances. Several vasoconstrictors and vasodilators are produced by the endothelium, such as endothelin-1, angiotensin-2, nitric oxide, and prostacyclin, which regulate vasomotor tone and the recruitment and activity of inflammatory cells and regulate thrombosis [20]. The normal balance between pulmonary vasodilators and vasoconstrictors is disrupted in ALI in favour of the latter, and thus results in increased pulmonary vascular resistance and pulmonary hypertension [21].

#### 3.2.1. Endothelin-1

Endothelin-1 (ET-1), a potent vasoconstrictor peptide produced by endothelial cells and degraded predominantly in the pulmonary vasculature, has long been implicated in the development of lung injury. ET-1 concentrations are elevated in ARDS as the result of both increased formation and decreased disposal [22]. In critically ill patients with sepsis, including ARDS subjects, increased endothelin production may contribute to local increases in vascular resistance, hypoperfusion, and the development of organ failure [23]. Patients with ARDS have increased plasma endothelin-1 levels, associated with abnormal pulmonary endothelin-1 metabolism. These abnormalities reverse in patients who recover [24]. It has been suggested that raised circulating ET-1 levels may partly contribute to the development of pulmonary vasoconstriction and bronchoconstriction associated with acute respiratory failure [25]. ET-1 has also been found in the lungs of subjects who died with ARDS, interestingly along with a decrease in both endothelial nitric oxide synthase and inducible nitric oxide synthase in the lung [26]. It has also been demonstrated that in patients with ARDS, ET-1 is produced mainly in the lung and is associated not only with pulmonary vasoconstriction but also the development of permeability oedema, leading to the impairment of oxygenation [27]. 

In experimental models of ARDS, ET-1 could contribute to pulmonary hypertension seen in acute lung injury [28], while the production of both ET-1 and nitric oxide (NO) was increased in serum and lung tissue in a VILI model [29]. ET-1 was released in an experimental model of oleic acid-induced lung injury [30]. ET-1 has been shown to be downregulated at a transcriptional and translational level by angiopoietin-1 (Ang-1) in both in vitro and in vivo systems, and moreover, cell-based Ang-1 gene transfer markedly ameliorated inflammation in vivo in an experimental model of ARDS. It was suggested that cell-based gene transfer of Ang-1 may provide a novel treatment strategy for ARDS by attenuating vascular inflammation via suppression of ET-1 [31].

Endothelin inhibitors and/or endothelin receptor blockade have also provided further evaluation for the involvement of ET-1 in lung injury and the use of ET-1 suppressors as potential treatments for inflammatory lung diseases. More specifically, phosphoramidon, an endothelin-converting enzyme inhibitor, attenuated LPS-induced ALI [32]; non-selective ET-1 receptor blockade by tezosentan attenuated lung injury in endotoxaemic sheep [33] and alpha-naphthylthiourea-induced lung injury in rats [34]; the P1/fl peptide that selectively antagonises endothelin-A receptors attenuated LPS-induced pulmonary NO production [35]; furthermore, the highly selective ET-1 receptor A inhibitor, sitaxentan, prevented BLM-induced pulmonary inflammation and fibrosis in a murine model [36].

#### 3.2.2. Renin–Angiotensin–Aldosterone System (RAAS)

Angiotensin converting enzyme (ACE), the key RAAS enzyme, is highly expressed on the surface of pulmonary microvascular EC [37]. ACE hydrolyses angiotensin I to angiotensin II and breaks down bradykinin, while its analogue, ACE2, converts angiotensin II into angiotensin (1–7). Angiotensin II exerts powerful vasoconstricting, pro-fibrotic, and pro-inflammatory effects, while angiotensin (1–7) is a potent vasodilator, anti-apoptotic, and anti-proliferative agent [38]. Therefore, ACE2 is considered a negative regulator of the classical ACE [39]. Results of both clinical and experimental studies have provided evidence for the implication of RAAS, and in particular of ACE, in the pathogenesis of acute lung injury. 

Clinical cohort studies have suggested the possible involvement of ACE in patients with ARDS. Plasma soluble ACE activity is decreased in ARDS patients [40] and the authors speculated that the decreased ACE levels in sepsis-induced ARDS are due to the presence of circulating inhibitors of ACE. Serum ACE levels were decreased and closely correlated with the severity of lung injury [41], while ACE2 activity was reduced in patients succumbing to ARDS [42]. Bronchoalveolar lavage fluid (BALF) ACE was elevated in ARDS patients with infectious causes of lung injury, possibly reflecting endothelial damage or local increase in ACE production in response to sepsis [43]. Thus, it has been suggested that the balance between ACE and ACE2 is crucial for controlling angiotensin II levels. ACE and ACE2 also appear to modify the severity of ARDS, with ACE2 playing a protective role [44]. A recent study has shown that the number of ACE-positive microvascular circulating endothelial microparticles (EMPs) were a prognostic marker for the development of ARDS in septic patients [45].

Various experimental studies have also demonstrated altered ACE activity in lung injury models. Angiotensin II induces pulmonary oedema in rabbits [46], while in a rat model of smoke inhalation-induced ARDS, inflammation pulmonary oedema and histological changes were possibly attributed to abnormal expression of ACE and ACE2 related pathway [47]. Another study has suggested that the reduced pulmonary microvascular endothelial ACE expression observed in septic ARDS is caused by a two-step process, involving an initially increased shedding of ACE followed by a compensatory downregulation of ACE mRNA and protein expression [48]. *ACE2* gene deletion worsens bleomycin-induced lung injury, whereas ACE2 protects against BLM-induced fibrosis. Hence, recombinant ACE2 may have therapeutic potential to reduce respiratory morbidity in ALI/ARDS [49]. ARDS is developed, in part, due to reduced pulmonary levels of angiotensin (1–7), and repletion of this peptide or an angiotensin II receptor antagonist can halt the development of ARDS [50]. It has also been reported that ACE2 and the angiotensin II type 2 receptor protect animals from severe acute lung injury induced by acid aspiration or LPS [51,52]. 

ACE2 was unexpectedly shown to act as the receptor for the severe acute respiratory syndrome (SARS) virus. It is now known that cells with increased expression of ACE2 have a higher probability to be infected by the new SARS coronavirus 2 (SARS-CoV2) also. Upregulation of ACE2 expression and function is increasingly recognized as a potential therapeutic strategy in hypertension and cardiovascular disease, diabetes, lung injury, and fibrotic disorders. Quantitative mRNA expression profiling of ACE2 showed expression in the thyroid and adrenal glands, and the pancreas [53,54].

The human *ACE* gene (*DCP1*) contains a restriction fragment length polymorphism within the coding sequence defined by the presence (insertion, I) or absence (deletion, D) of a 287-bp repeat. The human *ACE2* D allele confers increased ACE activity [55]. *ACE* I/D polymorphism has been shown to be associated with predisposition and prognosis in ARDS [56], while another study has shown that in ARDS patients, it acts as an independent risk factor for mortality [57]. A possible association between the *ACE* I/D polymorphism genotype and the mortality risk of ALI/ARDS in Asians has been also demonstrated [58], whereas in Chinese patients, the *ACE* I/D polymorphism is a significant prognostic factor for the outcome of ARDS, patients with the II genotype have a significantly better chance of survival; however, patients with the D allele do not have an increased risk for ARDS [59]. Other studies have failed to show any association. In paediatric ARDS, data did not support the association between *ACE* I/D genotype and ARDS, although severe hypoxemia was less frequent in D allele carriers, and *ACE* I/D polymorphism modified angiotensin-II levels [60]. Additionally, another study had data that did not support an association of the *ACE* gene I/D polymorphism with susceptibility or mortality in severe sepsis or with sepsis-induced ARDS in Spanish patients [61]. ACE activity may be the highest in patients with the DD genotype; however, its concentration and activity are also influenced by other mechanisms, in addition to the genotype; this might possibly explain the differing results regarding the *ACE* I/D polymorphism and ARDS susceptibility in the reports mentioned above.

The use of angiotensin II receptor blockers or ACE inhibitors has been shown to decrease lung injury in various animal models (reviewed in [62]); however, such a treatment in humans could lead to systemic hypotension [63]. Since ACE2 protects the lung from developing ARDS and functions as a coronavirus receptor for SARS [64], the recombinant ACE2 (rACE2) protein may have an important place in protecting ARDS patients and as a potential therapeutic approach in the management of emerging lung diseases [65]. 

#### 3.2.3. Pulmonary Endothelial ACE Activity as a Measure of Endothelial Function

Due to the very high enzyme concentrations in the capillaries, monitoring pulmonary endothelial ACE activity practically equals the monitoring of pulmonary capillary endothelium-bound (PCEB) ACE activity [66]. PCEB-ACE activity has been studied to a great extent in animal models and humans and has proven to comprise a quantifiable and sensitive measurement of endothelial function under normal conditions and lung diseases [5,7,67,68,69]. Pulmonary capillary ACE activity has been proven to be a reliable index of endothelial dysfunction in variable pulmonary diseases [70,71,72,73,74]. PCEB-ACE activity decreases early during ALI, which correlates with the clinical severity of both the lung injury and the underlying disease, and may be used as a quantifiable marker of underlying pulmonary capillary endothelial dysfunction [71].

#### 3.2.4. Endothelial Nitric Oxide Synthase (eNOS)

Nitric oxide (NO) is an effective vasodilator and inhibitor of vasoconstriction and platelet aggregation. Endothelial nitric oxide synthase (eNOS) is an enzyme abundantly expressed in the lung that constitutively produces nitric oxide (NO) [75]. Increased levels of eNOS-derived NO have been correlated with attenuated endotoxaemia, ventilator, ischemia-reperfusion and revascularization induced lung injury models [76,77,78,79,80,81,82,83,84]. On the other hand, animal studies seem to suggest that high bioavailability of NO from inducible NO synthase (iNOS) may worsen lung injury [85,86]. This might be explained by the fact that high or sustained NO levels may result in the formation of cytotoxic reactive nitrogen intermediates [87]. Furthermore, NO has been shown to rapidly react with oxygen in the lung forming nitrogen dioxide, a potent pulmonary irritant, and superoxide anion forming peroxynitrite, a cytotoxic oxidant that can lead to surfactant depletion [87]. Both iNOS and eNOS knock-out mice have been found to be more resistant to LPS-mediated increase in inflammatory cell infiltration, inflammatory cytokine production, and lung injury [88,89]. 

Therapeutic NO inhalation improves oxygenation in several ALI animal models and in responder ARDS patients, while in addition, it inhibits neutrophil activation, platelet adhesion, and the production of inflammatory mediators in the injured lungs [90,91,92,93,94]. However, subsequent clinical trials and meta-analyses have reported that inhaled nitric oxide resulted in a transient improvement in oxygenation but did not reduce mortality or duration of ventilator support in patients with ARDS, regardless of severity [95,96,97,98,99,100,101,102,103]. In some cases, it even seemed to be harmful, as it increased renal impairment [104,105]. Hence, it is suggested that the overall effect of inhaled nitric oxide in aggravating or attenuating inflammation and oxidative damage in injured lungs is dependent on its levels.

#### 3.2.5. Prostacyclin

Prostaglandins and thromboxanes are members of the group of biologically active lipid compounds called eicosanoids, and important mediators of inflammation. Prostaglandins include primary prostaglandins (PGE2, F2 alpha, D2) and prostacyclin (PGI2). Prostacyclin is a powerful vasodilator that inhibits the aggregation of blood platelets. In platelets, the actions of NO and prostacyclin are synergistic [106]. Fewer data are available for inhaled prostacyclin (iEPO). The most beneficial effects of iEPO have been seen in adult patients with severe ARDS, as demonstrated by the improvement in hemodynamic parameters and oxygenation [107,108,109,110]. A review originally published in 2010 and updated in 2017 concluded that there is no current evidence supporting or rebutting the routine use of aerosolised prostacyclin for ARDS patients [111].

### 3.3. Host Defence

The pulmonary endothelium is positioned between the vascular and lung airspaces, so it plays a critical role in establishing immune responses that lead to ARDS. ECs are key players in host defence and inflammation. Specifically, the lung endothelium provides the surface that joins inflammatory pathways of the innate immune system with the coagulation cascade [112]. Immune and inflammatory responses depend on communication between cells, which is mediated through cytokines, chemokines, interleukins, adhesion molecules, and growth factors. ECs produce and react to a variety of cytokines and other mediators [113,114]. The earliest work on ARDS biomarkers measured inflammatory cytokines in the BALF of “acute respiratory failure” patients. Interleukin-6 (IL-6), IL-8 and IL-10 have been the most widely studied cytokines to evaluate the intensity of the inflammatory response and help determine the prognosis for the patient. These markers also seem predictive of an adverse outcome in patients with localized infection and inflammation, such as in acute lung injury [115]. IL-6 is a prototype pro-inflammatory cytokine, IL-8 is a major chemokine, and IL-10 represents an important anti-inflammatory cytokine.

#### 3.3.1. Interleukins

##### IL-6

The study of inflammatory cytokines in the BALF of patients with ARDS has mainly shown persistent elevation over time with the prediction of poor outcome [116,117]. Plasma IL-6 levels correlate with ARDS development in intensive care unit (ICU) patients [118,119,120,121], but show variable results in trauma patients [122,123]. Other studies have also been able to confirm an inverse correlation between injurious ventilation and IL-6 levels [124,125]. An experimental study showed that IL-6 displays lung anti-inflammatory properties and exerts protective hemodynamic effects in a double-hit murine acute lung injury model [126]. Recently, it was shown that IL-6 levels in serum and BALF of an ARDS rat model were clearly increased compared with the normal control group [127].

##### IL-8

A study on IL-8 in BALF samples has also provided evidence of a relation between the presence of IL-8 in BALF and the development of ARDS. The authors suggested that the early appearance of IL-8 in BALF of patients at risk of ARDS may be an important prognostic indicator for the development of the disorder [128]. Likewise, plasma IL-8 levels have been shown to correlate with ARDS development in ICU and trauma patients [119,122,123]. A combination of biomarkers that include IL-8 has been shown to be superior to clinical predictors or single biomarkers for predicting mortality in ARDS [129,130,131]. Very recently, it was shown that plasma IL-8 levels were associated with clinical outcomes such as mortality, but not associated with paediatric ARDS development [132]. 

A monoclonal antibody against IL-8 has shown protective effects in lung injury caused by various insults, presenting a new hope for the prevention and treatment of ARDS [133,134,135,136].

##### IL-10

In VILI experimental models, IL-10 has been shown to regulate the inflammatory response in the lung tissue, improve lung tissue oxygenation, inhibit oxidative stress and reduce biotrauma and mortality, revealing a potential new treatment option for VILI [137,138,139]. Plasma cytokines IL-6, IL-8, and IL-10 are associated with ARDS in patients with severe traumatic brain injury [140]. Moreover, plasma biomarkers measured at the onset of acute lung injury, when combined with clinical data, can improve prognosis accuracy [129,131].

Genetic variability in pro- and anti-inflammatory cytokines has been suggested to contribute to different clinical phenotypes in patients at high risk of critical illness, including ARDS [141]. 

#### 3.3.2. Leukocytes and Pulmonary Endothelium

Activation and transmigration of circulating polymorphonuclear leukocytes (PMNs) play a major role in the early development of ALI [142]. Transendothelial migration of leukocytes is required for normal host defence, vasomotor tone, and inflammation. Persistent opening, however, of the intercellular junctions leads to the formation of protein-rich oedema in the interstitial tissue, which can lead to life-threatening illness, such as ARDS [16]. The activated neutrophils transmigrate into lung tissue across the endothelium to sites of pathogen invasion or tissue damage due to a toxic or physical insult. Apart from the beneficial roles of PMNs at the inflamed sites, production of reactive oxygen species (ROS) and secretion of neutrophil extracellular traps (NETs), also confer to PMNs auto-damaging functions [143]. The overproduction of NETs has been shown to be involved in the inflammatory injury of lung tissues [144,145,146,147].

##### The Selectin Family

The selectin family is an early mediator of the adhesion of activated PMNs to endothelial cells in inflammatory states, before their firm adhesion and diapedesis at sites of tissue injury and inflammation [148]. The initial steps of the leukocyte adhesion cascade include capture and rolling of circulating leukocytes and require E-, L and P-selectin [149]. Ligands of the selectin family, comprising L-selectin (expressed on leukocytes), E-selectin (expressed on ECs) and P-selectin (ECs and platelets), tether circulating leukocytes reversibly on the EC surface and facilitate rolling of leukocytes along the endothelium to the point of transmigration. Selectins are a family of adhesion molecules implicated in leukocyte-endothelial adhesion; whose receptors can exist in a soluble (s) form. 

Plasma levels of sE- and sP-selectin have been found to be elevated among ARDS patients [150,151,152]. Another study has shown that determination of sE-selectin levels might be useful for prediction of the development of ARDS in critically ill patients [153] and furthermore, ARDS patients with alcohol abuse problems have elevated concentrations of plasma sE-selectin [154]. Elevated sE-selectin levels were found in patients with established ARDS, raising the possibility that sE-selectin exerts pro-inflammatory effects upon neutrophil function at sites of inflammation, thereby aggravating disease processes [155]. Plasma sE-selectin was also a good predictor biomarker for both mechanical ventilation duration and mortality risk in children with ARDS [156].

Treatment, however, with antibodies against selectins has shown conflicting results, depending on the type of lung injury. An antibody to E- and L-selectin did not protect against Gram-negative sepsis-induced lung injury in primates [157], while a dual-binding antibody to E- and L-selectin attenuated sepsis-induced lung injury in swine [158]. An anti-P-selectin antibody failed to protect against lung injury; however, it decreased some aspects of injury in the peripheral microcirculation in sheep with burn and smoke-induced lung injury [159]. Other studies, on the other hand, were able to show protective effects by blocking P-selectin with antibodies in LPS-induced lung injury [160], in cobra venom factor-induced lung injury [161], and in acid-aspiration-induced lung injury [162].

Recently, the selectin P ligand gene (*SELPLG*) was identified as a novel ARDS susceptibility gene among individuals of European and African descent, and furthermore an antibody that neutralizes P-selectin glycoprotein ligand 1 significantly attenuated LPS-induced lung inflammation [163]. 

Since leukocyte migration into the tissues is a key process in the pathogenesis of inflammatory diseases and activation of circulating neutrophils and transmigration into the alveolar airspace are associated with the development of ARDS, inhibitors of neutrophil recruitment may attenuate lung damage [164].

##### Soluble Intercellular Adhesion Molecule-1 (sICAM-1)

Intercellular adhesion molecule-1 (ICAM-1) controls the firm adhesion of neutrophils on the endothelium and facilitates their transendothelial migration via the platelet-endothelial cell adhesion molecule-1 (PECAM-1). Similar to the selectin family mentioned above, elevated soluble ICAM-1 levels have been observed in the serum of patients with ARDS or those who are at risk of developing ARDS [165,166,167], while immunohistochemical investigations have demonstrated the induction of ICAM-1 in human ARDS lungs [168].

In animal models, the central role of ICAM-1 in ARDS has been extensively studied [169]. To this end, various compounds have been successful in ameliorating lung inflammation in animal models of ARDS by reducing endothelial expression of ICAM-1 [170,171,172,173].

### 3.4. Haemostasis and Coagulation

Inflammation and coagulation are critical host responses to infection and injury and are involved in ARDS pathogenesis. It has been recognized that ECs coordinate the immune and haemostatic response by shifting from their normal anti-thrombotic and anti-inflammatory phenotype to an activated state of endothelial dysfunction [174]. ECs actively regulate haemostasis by producing a variety of proteins, which include pro-thrombotic substances (von Willebrand factor, P-selectin), molecules restricting coagulation (heparan sulphate, thrombomodulin, NO, prostacyclin, tissue factor pathway inhibitor) and fibrinolytic factors (plasminogen activators) [175]. 

#### 3.4.1. von Willebrand Factor (vWf)

Key events leading to the activated state of the endothelium include the expression of adhesion molecules to leukocytes and platelets on the EC surface in addition to the expression of activators of the humoral clotting system, including tissue factor and von Willebrand factor (vWf) [176,177]. Numerous studies have shown that vWF is altered in ALI/ARDS and that it is a sensitive marker indicating EC injury or activation [129,131]. For example, plasma vWf levels are predictive of the development of ARDS and denote poor prognosis in patients following severe trauma [178], while higher levels have been detected in non-survivors [179,180]. Moreover, increased concentrations of the A2 domain of vWf in the first 24 h post admission in burn-injured patients were strongly associated with the development of ARDS [181]. In patients with non-pulmonary sepsis, elevated plasma vWf levels may be used as an early marker of endothelial damage with both predictive and prognostic value [182]. vWf levels also seem to correlate with ARDS mortality in paediatric patients [183]. However, in heterogeneous cohorts of “at risk” patients, vWf levels are not predictive for ARDS [184,185,186]. The biology and prognosis of ARDS differ between direct and indirect mechanisms of lung injury and the contrasting findings of the association of vWF and ARDS in the studies discussed above can be attributed to diverse study cohorts [187].

In experimental animal models, plasma vWF appears to be considerably increased prior to significant damage to the endothelium [176].

#### 3.4.2. Coagulation

The coagulation system is a major participant in ARDS [188]. Enhanced activation of coagulation combined with dysfunction of the anti-coagulant mechanisms constitute both consequences of and contributors to ongoing lung injury. Upon vascular injury, the haemostatic system initiates a series of vascular events, which result in activation of extravascular receptors that act to seal off the damage by increasing coagulation and depressing fibrinolysis [189]. Tissue factor (TF) is the major initiator of the extrinsic coagulation pathway. TF binds and activates factor VII, and the TF-activated factor VII (FVIIa) generated complex triggers coagulation by activating factor X. Activated factor X (FXa) allows conversion of prothrombin to thrombin [190]. Conversely, the protein C (PC) system provides important control of coagulation by virtue of the capacity of activated protein C (APC) to proteolytically inactivate the cofactors Va and VIIIa. APC is generated by thrombomodulin (TM)-bound thrombin [191]. The endothelial protein C receptor (EPCR) facilitates this activation. The membrane-bound EPCR regulates the protein C anticoagulant and anti-inflammatory pathways, while its soluble form (sEPCR) inhibits APC activities and has been implicated in sepsis [192]. TM is an anticoagulant proteoglycan located on the EC surface, which reacts with thrombin producing a marked increase in the thrombin-catalysed activation of protein C; hence, thrombin also has anticoagulant properties in addition to pro-coagulant properties [7,193,194]. While the pro-coagulant protein thrombin disrupts inter-endothelial junctions, the anti-coagulant APC enhances the vascular barrier [195,196].

In ARDS, alveolar thrombin generation seems to be mediated by the TF pathway, which is extensively activated in such patients [197]. Patients who develop ventilator-associated pneumonia (VAP) have increased BALF levels of soluble TF and FVII, and moreover, increased BALF levels of soluble TF, FVIIa and FXa have been demonstrated in patients with ARDS [115]. Inhibition of the TF-FVIIa pathway completely abolished intrapulmonary fibrin deposition in ARDS patients [198], attenuated lung injury and prevented local activation of coagulation in models of pneumonia [199]. Pro-coagulant activity in the form of factor X activating activity has been observed in the BALF of adult patients with ARDS [200]. 

Altered plasma levels of PC, TM and EPCR are associated with poor clinical outcomes in patients with ARDS. Apart from a reduction in APC levels, soluble levels of TM in pulmonary oedema fluid from patients with ARDS are significantly higher than those in plasma [201]. The PC system is markedly disrupted in patients with ARDS and plasma TM is increased in ARDS patients, possibly through proteolytic release from the injured pulmonary endothelium [202,203]. Similarly, plasma TM is increased in infants with respiratory distress syndrome, especially in those treated with mechanical ventilation [204], while elevated plasma soluble (s)TM levels were associated with organ dysfunction in children with ARDS and with higher mortality in children with indirect lung injury [205]. Soluble TM in BALF was found to be an independent predictor of severe drug-induced lung injury [206], and moreover, higher plasma sTM levels were associated with increased mortality in ARDS [207]. Protein C has also been found to be an independent predictor of mortality for ALI/ARDS [130,208]. Mice with malaria-associated ARDS showed an increase in EPCR concentrations in lung homogenates [209], whereas loss of EPCR and TM was seen in lung specimens from patients who died from severe falciparum malaria with coexisting ARDS [210].

Genetic variants in the *TM* and *EPCR* genes have been demonstrated to be additively associated with mortality in ARDS, suggesting that genetic differences may be at least partially responsible for the observed associations between dysregulated coagulation and poor outcomes in ARDS [211].

#### 3.4.3. Fibrinolysis

The pulmonary endothelium is actively involved in the fibrinolytic process, expressing, amongst others, plasminogen activator inhibitors [193]. Plasminogen activator inhibitor-1 (PAI-1) is the major inhibitor of fibrinolysis, whose upregulation leads to a shift from pro- to anti-fibrinolytic phenotypes [212]. Several studies have shown an increase in serum levels in patients with ARDS [131,213]. Additionally, human pulmonary microvascular endothelial cells (PMECs) isolated from ARDS patients expressed higher pro-coagulant activity and PAI-1, and lower fibrinolytic potential compared to controls, confirming the pro-coagulant properties of the pulmonary endothelium in ARDS [214]. Increased plasma concentrations of TF and PAI-1 might support ARDS diagnoses in mechanically-ventilated patients [215], and alveolar PAI-1 predicts ARDS in aspiration pneumonitis [216]. In another study, PAI-1 levels in pulmonary oedema fluid and plasma were able to identify ARDS patients who have a poor prognosis [217], while enhanced pro-coagulant and depressed fibrinolytic activities were found in the BALF of patients with ARDS [218]. In addition, patients who developed VAP showed a significant increase in pro-coagulant activity with concomitant depressed fibrinolytic activity [219].

Coagulation (as measured by plasma levels of protein C) and fibrinolysis (as measured by plasma levels of PAI-1) have been shown to be markedly abnormal in ARDS and independently associated with adverse clinical outcomes; this pro-coagulant, anti-fibrinolytic phenotype was present regardless of the underlying cause of lung injury [208]. Coagulopathy and alveolar epithelial injury have been also observed in patients with direct common risk factors (direct ARDS) and those with idiopathic or immune-related diseases (indirect ARDS); however, their biomarker profiles were significantly different [220].

Regulation of coagulation and thrombolysis and promotion of haemofluidity aid in maintaining rapid and unobstructed blood flow. Targeting these pathways has been a focus of therapy-based approaches, with limited success. In patients with ARDS, combined treatment with sivelestat, an inhibitor of human neutrophil elastase, and recombinant human TM (rhTM) had beneficial effects on survival [221]. Data from murine experimental models have suggested that recombinant TM (rTM) may have a potential therapeutic effect for surgical ARDS via suppression of the secretion of pro-inflammatory cytokines [222], while in a murine model of severe ARDS, rTM administration prolonged the survival time and ameliorated the development of ARDS [223]. In two murine models of LPS and LPS + VILI, single therapy with anti-thrombin did not attenuate the pronounced pulmonary coagulation or inflammatory response [224].

#### 3.4.4. Platelet–EC Interaction

Platelets prevent blood loss by forming the platelet haemostatic plug. They also serve as a platform for coagulation factors. The interaction of the endothelium with platelets possesses a central part in their activation and regulation; an intact endothelium prevents the adhesion of platelets, while activated ECs express molecules and receptors that promote platelet adhesion to the injury site. Although platelets induce lung inflammation leading to ARDS, the extent of platelet–EC interactions remains poorly understood. Furthermore, platelets also interact with immune cells, promoting haemostasis and inflammation [225]. Data from animal and human studies support the important role of platelet–neutrophil interaction in ARDS immunothrombosis. In this review, we will not deal with platelet interactions, but will refer the readers to a recent and thorough review [188].

### 3.5. Angiogenesis

Vascular development strongly depends on the collaboration of growth factors. Studies have implicated vascular endothelial growth factors (VEGFs), angiopoietins, and ephrins as critical players in particular aspects of vascular development [226]. 

#### 3.5.1. VEGF

Vascular endothelial growth factor (VEGF) is a glycoprotein originally isolated as a permeability factor with unique specificity for vascular ECs [227], but was subsequently shown to have mitogenic and angiogenic properties [228]. 

The majority of research investigating the role of VEGF in the lung has focused on the VEGF-A molecule. To date, most studies of lung injury in humans show a reduction in intrapulmonary VEGF levels in the early stages of ARDS [229]. Maitre et al. showed that the initial phase of ARDS is associated with a decrease in VEGF in the lung and they suggested that this down-regulation may represent a protective mechanism by limiting endothelial permeability [230]. In a separate study, the possible role of decreased VEGF levels in lowering lung perfusion in ARDS was demonstrated [231], while Azamfirei et al. showed that the initial phase of ARDS is associated with a decrease in VEGF in the lung with a simultaneous increase in the plasma. They also reported that persistent elevation of plasma VEGF over time predicts poor outcome [232]. The VEGF receptors 1 and 2 (VEGFR1 and VEGFR2, respectively) were significantly up-regulated in later ARDS compared to normal subjects and early ARDS; this up-regulation of VEGFR1 and VEGFR2 in late ARDS suggests regulation of VEGF bioactivity by its receptors, assigning a protective role to VEGF in lung injury recovery [233]. Another human study was able to show the presence of soluble VEGFR1 in the BALF of ARDS patients, possibly explaining the reduced levels of bioactive VEGF in early ARDS [234]. A different study showed increased plasma VEGF levels in ARDS patients compared to normal control subjects. The increased plasma VEGF levels were associated with mortality [235]; these increased levels are thought to arise from the VEGF “pool” contained in activated neutrophils [236] and macrophages [237]. Moreover, the latter study has demonstrated that initially increased VEGF levels in the alveolar space in ARDS patients are reduced in recovery; the authors suggested that VEGF in the alveolar space may reflect the exudative and repair phase of ARDS, as opposed to plasma levels [237]. The VEGF-B molecule has also been found to be decreased in ARDS, suggesting a role in repair after lung injury [238].

Variations in plasma VEGF levels might also be attributed to the several polymorphisms reported for the *VEGF* gene. Specifically, lower VEGF plasma levels have been linked to the presence of the T allele in the +936 CT polymorphism. Hence, several studies have hypothesized that the presence of the T allele might be associated with the development and severity of ARDS. Indeed, the CT and TT genotype frequencies were increased in ARDS patients compared with normal subjects [239], while they were also significantly associated with increased mortality and contributed to the prognosis and inter-individual variations in circulating VEGF levels in patients with ARDS [240]. Another study has demonstrated that the *VEGF* +936 TT genotype is a risk factor and may contribute to the prognosis of ARDS in the Chinese population [241]. Another possible explanation for the reduction in intrapulmonary VEGF in ARDS subjects includes alternative splicing of the *VEGF* gene, yielding soluble and membrane-bound isoforms. Indeed, the ratio of soluble to membrane-bound isoforms is lower in early ARDS compared to healthy subjects and later ARDS [242].

VEGF levels in serum and BALF of an ARDS rat model were increased compared with the normal control group [127]. In another study, fat embolism (FE)-induced acute lung injury significantly increased pulmonary VEGF expression; furthermore, systemic administration of a VEGFR2 antagonist significantly attenuated the FE-induced inflammatory response and histological damage [243]. Kaner et al. showed that overexpression of *VEGF* in murine lung may represent one mechanism of increased pulmonary vascular permeability in the early stages of ALI. In the same study, pre-treatment with recombinant VEGFR1 completely abrogated the increased vascular permeability [244]. Similarly, pre-treatment with VEGFR2 prevented ischemia-reperfusion induced lung injury in rats [245]. Bevacizumab (an anti-VEGF antibody) attenuated oedema caused by high permeability [246], while instillation of VEGF via anti-VEGFR2 antibodies protected mice against respiratory distress syndrome [247]. 

#### 3.5.2. Angiopoietin-2 (Ang-2)

Angiopoietins (Angs) are a novel class of angiogenic growth factors, which have been implicated in the pathophysiology of sepsis and ARDS. Ang-1 is a Tie2 receptor agonist that induces endothelial migration, inhibits endothelial apoptosis, reduces vascular permeability and inflammation, maintains vascular integrity, and has diverse vasoprotective and anti-inflammatory actions. In contrast, Ang-2 disrupts the protective effects of Ang-1-Tie2 signalling [248,249]. 

The release of Ang-2 has been shown to directly reflect vascular barrier breakdown [250,251,252]. Circulating Ang-2 has been correlated with mortality in a surgical population with ARDS [253], and with pulmonary permeability oedema, incidence and severity of ARDS in patients with and without sepsis [254]. Plasma Ang-2 predicts the onset of ARDS in critically ill patients [186], while increased pulmonary vascular permeability and inflammation due to Ang-2 have been suggested to play a role in the pathogenesis of idiopathic interstitial pneumonia [255]. The plasma levels of Ang-2 were significantly increased in patients with ARDS [152] and have been correlated with the severity of lung injury [256]. Moreover, plasma Ang-2 has been shown to surpass other markers of endothelial injury in prognosticating paediatric ARDS mortality [257], while it was also shown that increased Ang-2 levels in the alveolar compartment of ARDS patients were associated both with increased mortality and failure in other organs in addition to the lung [258]. In ARDS not related to infection, higher baseline Ang-2 levels were strongly associated with increased mortality [259]. Recent studies have demonstrated that increased Ang-2 levels early after admission are significantly associated with the risk of mortality in sepsis patients with concomitant ARDS [260], which could be a causal factor in sepsis-associated ARDS development [261], and could also predict severe acute kidney injury (AKI), specifically in ARDS patients [262]. Finally, a very recent systematic review and meta-analysis of 10 prospective cohort studies has shown that higher circulating Ang-2 levels constituted independent predictors of the risk of mortality in patients with ARDS [263].

Genetic variants in the *Ang-2* gene have also been associated with increased risk of ARDS [264], while soluble receptors of angiogenic factors, namely sVEGFR2, are considered to be valuable predictive biomarkers in the development of ARDS associated with critical illness and mortality in such patients [265]. Biomarker panels that include Ang-2 have also performed well across multiple patient cohorts and have outperformed clinicians’ diagnosis of ARDS in severe trauma [266]. Thus, such panels could improve both diagnosis and treatment of ARDS [152,266,267].

Synthetic Tie-2 agonists have been shown to protect against vascular leakage in murine sepsis models and activate the receptor and its downstream pathways [268,269]. Therefore, it has been suggested that targeting the Tie-2 receptor may be important in treatment of pathological vascular leakage. Other data have demonstrated that selective blockade of the Ang-2 function decreased lung protein leak and indices of inflammation, and improved survival in an ARDS murine model [270], while lung-targeted RNA interference of Ang-2 ameliorated multiple organ dysfunction and mortality in sepsis [271].

Hence, inhibiting Ang-2 may represent a potential anti-inflammatory and anti-vascular hyper-permeability strategy in the treatment of sepsis and ARDS [272]. 

### 3.6. COVID-19 and ARDS

A novel coronavirus, SARS-CoV-2, caused the outbreak of an unknown infectious pneumonia, which is now termed coronavirus disease-19 (COVID-19) [273]. The global pandemic of COVID-19 is associated with the development of ARDS, requiring ventilation in critically ill patients. COVID-19 has been associated with ischemic complications, coagulation disorders, and endothelitis. ACE2 is thought to be a primary mechanism of SARS-CoV-2 entry and infection [274]. This could possibly provide a mechanism for the vascular thrombosis seen in COVID-19 patients. [274,275].

The histologic pattern in the peripheral lung of non-survivors with COVID-19-associated respiratory failure was diffuse alveolar damage with perivascular T-cell infiltration. The lungs of the COVID-19 patients also showed distinct vascular features, including severe endothelial injury and disrupted cell membranes. Most importantly, vascular angiogenesis could discriminate the pulmonary pathobiology of COVID-19 from that of influenza infection [276].

A characteristic feature of COVID-19 patients is elevated D-dimers and fibrinogen. The fibrin deposits found in the patients’ lungs are most possibly due to dysregulation of coagulation and fibrinolysis. Damaged endothelial cells and leukocytes expose TF, promoting fibrin deposition, while increased PAI-1 levels from lung epithelium and ECs promote a hypo-fibrinolytic state. Hence, nebuliser plasminogen activators may provide a treatment therapy for COVID-19 patients, by facilitating fibrin degradation and improving oxygenation [213].

A recent study has demonstrated that Ang-2 could be a predictive marker for ICU admission in COVID-19 patients; this endothelial activation seen in COVID-19 further supports the hypothesis of a COVID-19-associated microvascular dysfunction [277]. 

A common complication of COVID-19 is the phenomenon of a “cytokine storm”. Cytokine-driven vascular leak in the lung alveolar-endothelial interface promotes acute lung injury in the setting of viral infection. This hyperinflammatory response results in diffuse alveolar damage [278]. Furthermore, two main pathomechanisms preceding COVID-19-associated severe respiratory failure have been proposed: (1) macrophage activation syndrome, and (2) IL-6-driven defective antigen-presentation [279]. Thus, molecules are being tested to improve the clinical outcomes of COVID-19 by attenuating hyperinflammation and immune dysregulation, and the potential development of a cytokine storm. Ciceri et al. have actually suggested a new name for the disease, the use of microvascular COVID-19 lung vessels obstructive thromboinflammatory syndrome (MicroCLOTS). They hypothesise that, in predisposed individuals, alveolar viral damage is followed by an inflammatory reaction and by microvascular pulmonary thrombosis [280].

Given that the virus affects multiple organs, including the heart, it likely gains access into systemic circulation by infecting or passing from the respiratory epithelium to the endothelium for viral dissemination. Indeed, cardiovascular complications of COVID-19 are highly prevalent and include acute cardiac injury, myocarditis, and a hypercoagulable state, all of which may be influenced by altered endothelial function [278]. 

Goshua et al. suggested that endotheliopathy and platelet activation are important features of COVID-19 in hospitalised patients and are likely to be associated with critical illness and death. Hence, they propose that early identification of endotheliopathy and strategies to mitigate its progression might improve outcomes in COVID-19 [281].

Rapidly emerging data on COVID-19 are providing insight into how endothelial dysfunction may contribute to the pandemic. This may lead to the search for prognostic biomarkers, as well as therapeutics targeting pathogenic endothelial responses.

## 4. Conclusions

The pulmonary endothelium is a major component of the alveolar-capillary unit, rendering it vulnerable to injury from various insults (mechanical, chemical, or cellular) that are either inhaled or delivered to the lung through pulmonary circulation. The most severe form is acute respiratory distress syndrome (ARDS), which is associated with high morbidity and mortality rates. ARDS is caused by an unregulated, auto-destructive inflammatory process characterized by the activation of intrapulmonary and circulating cells, as well as the invasion of neutrophils and cytokine production, resulting in the breakdown of the lung barrier and gas exchange functions. ARDS pathogenesis has not been completely elucidated; however, it is known that the pulmonary endothelium plays a major role by altering haemostasis, weakening barrier function and mediating intercellular signalling [7]. The overall result in the lung is a clinical picture of increased alveolar oedema with diffuse infiltrates, respiratory distress, refractory hypoxemia, and respiratory failure [1,193,282]. Thus, understanding the pathophysiological mechanisms leading to this disorder may give insights into future therapies.

## Figures and Tables

**Table 1:** Major pulmonary endothelial functions.

Barrier and transport functions
Synthesis of vasoactive compounds–maintenance of vascular tone
Host defence—production of cytokines and chemokines
Haemostasis and coagulation
Angiogenesis—production of growth factors
Expression of receptors and signal transduction molecules
Expression of adhesion molecules
Production of reactive oxygen species

## Abbreviations

ARDS	acute respiratory distress syndrome
COVID-19	coronavirus disease 19
ALI	acute lung injury
VILI	ventilator-induced lung injury
LPS	lipopolysaccharide
BLM	bleomycin
EC	endothelial cell
IEJ	interendothelial junction
TJ	tight junction
AJ	adherens junction
GJ	gap junction
JAM	junctional adhesion molecule
VE-cadherin	vascular endothelial cadherin
LMVEC	lung microvascular endothelial cell
EG	endothelial glycocalyx
ET-1	endothelin-1
NO	nitric oxide
Ang-1	angiopoietin-1
RAAS	renin–angiotensin–aldosterone system
ACE	angiotensin converting enzyme
BALF	bronchoalveolar lavage fluid
EMP	endothelial microparticle
SARS	severe acute respiratory syndrome
SARS-CoV-2	severe acute respiratory syndrome coronavirus 2
rACE2	recombinant ACE2
PCEB	pulmonary capillary endothelium-bound
eNOS	endothelial nitric oxide synthase
iNOS	inducible nitric oxide synthase
iEPO	inhaled prostacyclin
IL	interleukin
ICU	intensive care unit
PMN	polymorphonuclear cell
ROS	reactive oxygen species
NET	neutrophil extracellular trap
sE-selectin	soluble E-selectin
sP-selectin	soluble P-selectin
sICAM-1	soluble intercellular adhesion molecule-1
PECAM-1	platelet-endothelial cell adhesion molecule-1
vWF	von Willebrand factor
TF	tissue factor
PC	protein C
APC	activated protein C
TM	thrombomodulin
EPCR	endothelial protein C receptor
VAP	ventilator-associated pneumonia
PAI-1	plasminogen activator inhibitor 1
PMEC	pulmonary microvascular endothelial cell
rhTM	recombinant human TM
rTM	recombinant TM
VEGF	vascular endothelial growth factor
VEGFR1	VEGF receptor 1
VEGFR2	VEGF receptor 2
FE	fat embolism
AKI	acute kidney injury
COVID-19	coronavirus disease-19
MicroCLOTS	microvascular COVID-19 lung vessels obstructive thromboinflammatory syndrome

## References

[1] Force A.D.T., Ranieri V.M., Rubenfeld G.D., Thompson B.T., Ferguson N.D., Caldwell E., Fan E., Camporota L., Slutsky A.S. (2012). Acute Respiratory Distress Syndrome. JAMA.

[2] Martin T.R. (1999). Lung Cytokines and ARDS. Chest.

[3] Bernard G.R., Artigas A., Brigham K.L., Carlet J., Falke K., Hudson L., Lamy M., Legall J.R., Morris A., Spragg R. (1994). The American-European Consensus Conference on ARDS. Definitions, mechanisms, relevant outcomes, and clinical trial coordination. Am. J. Respir. Crit. Care Med..

[4] Sweatt A.J., Levitt J.E. (2014). Evolving Epidemiology and Definitions of the Acute Respiratory Distress Syndrome and Early Acute Lung Injury. Clin. Chest Med..

[5] Maniatis N.A., Kotanidou A., Catravas J.D., Orfanos S.E. (2008). Endothelial pathomechanisms in acute lung injury. Vasc. Pharmacol..

[6] Maniatis N.A., Orfanos S.E. (2008). The endothelium in acute lung injury/acute respiratory distress syndrome. Curr. Opin. Crit. Care.

[7] Orfanos S.E., Mavrommati I., Korovesi I., Roussos C. (2004). Pulmonary endothelium in acute lung injury: From basic science to the critically ill. Intensive Care Med..

[8] Mehta D., Malik A.B. (2006). Signaling Mechanisms Regulating Endothelial Permeability. Physiol. Rev..

[9] Galley H.F., Webster N.R. (2004). Physiology of the endothelium. Br. J. Anaesth..

[10] Matute-Bello G., Frevert C.W., Martin T.R. (2008). Animal models of acute lung injury. Am. J. Physiol. Cell. Mol. Physiol..

[11] Vandenbroucke E., Mehta D., Minshall R., Malik A.B. (2008). Regulation of endothelial junctional permeability. Ann. N. Y. Acad. Sci..

[12] Komarova Y., Malik A.B. (2010). Regulation of Endothelial Permeability via Paracellular and Transcellular Transport Pathways. Annu. Rev. Physiol..

[13] Sukriti S., Tauseef M., Yazbeck P., Mehta D. (2014). Mechanisms Regulating Endothelial Permeability. Pulm. Circ..

[14] Matthay M.A., Zemans R.L., Zimmerman G.A., Arabi Y.M., Beitler J.R., Mercat A., Herridge M., Randolph A.G., Calfee C.S. (2019). Acute respiratory distress syndrome. Nat. Rev. Dis. Primers.

[15] Rippe B., Rosengren B.-I., Carlsson O., Venturoli D. (2002). Transendothelial Transport: The Vesicle Controversy. J. Vasc. Res..

[16] Tsushima K., King L.S., Aggarwal N.R., De Gorordo A., D’Alessio F.R., Kubo K. (2009). Acute Lung Injury Review. Intern. Med..

[17] Liu Y., Mu S., Li X., Liang Y., Wang L., Ma X. (2019). Unfractionated Heparin Alleviates Sepsis-Induced Acute Lung Injury by Protecting Tight Junctions. J. Surg. Res..

[18] Benatti M.N., Fabro A.T., Miranda C.H. (2020). Endothelial glycocalyx shedding in the acute respiratory distress syndrome after flu syndrome. J. Intensive Care.

[19] Zhang D., Qi B.-Y., Zhu W.W., Huang X., Wang X. (2020). Crocin alleviates lipopolysaccharide-induced acute respiratory distress syndrome by protecting against glycocalyx damage and suppressing inflammatory signaling pathways. Inflamm. Res..

[20] Epstein F.H., Vane J.R., Änggård E.E., Botting R.M. (1990). Regulatory Functions of the Vascular Endothelium. N. Engl. J. Med..

[21] Moloney E.D., Evans T.W. (2003). Pathophysiology and pharmacological treatment of pulmonary hypertension in acute respiratory distress syndrome. Eur. Respir. J..

[22] Druml W., Steltzer H., Waldhäusl W., Lenz K., Hammerle A., Vierhapper H., Gasic S., Wagner O.F. (1993). Endothelin-1 in Adult Respiratory Distress Syndrome. Am. Rev. Respir. Dis..

[23] Sanai L., Haynes W.G., MacKenzie A., Grant I.S., Webb D.J. (1996). Endothelin production in sepsis and the adult respiratory distress syndrome. Intensive Care Med..

[24] Langleben D., DeMarchie M., Laporta D., Spanier A.H., Schlesinger R.D., Stewart D.J. (1993). Endothelin-1 in acute lung injury and the adult respiratory distress syndrome. Am. Rev. Respir. Dis..

[25] Mitaka C., Hirata Y., Nagura T., Tsunoda Y., Amaha K. (1993). Circulating Endothelin-1 Concentrations in Acute Respiratory Failure. Chest.

[26] Albertine K.H., Wang Z.M., Michael J.R. (1999). Expression of endothelial nitric oxide synthase, inducible nitric oxide synthase, and endothelin-1 in lungs of subjects who died with ARDS. Chest.

[27] Nakano Y., Tasaka S., Saito F., Yamada W., Shiraishi Y., Ogawa Y., Koh H., Hasegawa N., Fujishima S., Hashimoto S. (2007). Endothelin-1 level in epithelial lining fluid of patients with acute respiratory distress syndrome. Respirology.

[28] Pritze S., Peskar B.A., Simmet T. (1992). Release of eicosanoids and endothelin in an experimental model of adult respiratory distress syndrome. Agents Actions Suppl..

[29] Lai T.-S., Cai S.-X., Guo Z.-H. (2010). Serum and lung endothelin-1 increased in a canine model of ventilator-induced lung injury. Chin. Med. J..

[30] Simmet T., Pritze S., Thelen K.I., Peskar B.A. (1992). Release of endothelin in the oleic acid-induced respiratory distress syndrome in rats. Eur. J. Pharmacol..

[31] McCarter S.D., Lai P.F.H., Suen R.S., Stewart D.J. (2006). Regulation of endothelin-1 by angiopoietin-1: Implications for inflammation. Exp. Biol. Med..

[32] Bhavsar T.M., Cerreta J.M., Liu M., Reznik S.E., Cantor J.O. (2008). Phosphoramidon, an endothelin-converting enzyme inhibitor, attenuates lipopolysaccharide-induced acute lung injury. Exp. Lung Res..

[33] Kuklin V., Kirov M., Sovershaev M., Andreasen T., Ingebretsen O.C., Ytrehus K., Bjertnaes L.J. (2005). Tezosentan-induced attenuation of lung injury in endotoxemic sheep is associated with reduced activation of protein kinase C. Crit. Care.

[34] Atalay F., Yurdakan G., Yilmaz-Sipahi E. (2012). Effect of the endothelin receptor antagonist tezosentan on alpha-naphthylthiourea-induced lung injury in rats. Kaohsiung J. Med. Sci..

[35] Fujii Y., Magder S., Cernacek P., Goldberg P., Guo Y., Hussain S.N.A. (2000). Endothelin Receptor Blockade Attenuates Lipopolysaccharide-induced Pulmonary Nitric Oxide Production. Am. J. Respir. Crit. Care Med..

[36] Manitsopoulos N., Nikitopoulou I., Maniatis N.A., Magkou C., Kotanidou A., Orfanos S.E. (2018). Highly Selective Endothelin-1 Receptor A Inhibition Prevents Bleomycin-Induced Pulmonary Inflammation and Fibrosis in Mice. Respiration.

[37] Aird W.C. (2007). Phenotypic heterogeneity of the endothelium: II. Representative vascular beds. Circ. Res..

[38] Pagliaro P., Penna C. (2020). ACE/ACE2 Ratio: A Key Also in 2019 Coronavirus Disease (Covid-19)?. Front. Med..

[39] Chambers S., Bhatia M. (2014). ACE and ACE2 in Inflammation: A Tale of Two Enzymes. Inflamm. Allergy Drug Targets.

[40] Casey L., Krieger B., Kohler J., Rice C., Oparil S., Szidon P. (1981). Decreased serum angiotensin converting enzyme in adult respiratory distress syndrome associated with sepsis. Crit. Care Med..

[41] Fourrier F., Chopi C., Wallaert B., Mazurier C., Mangalahoyi J., Durocher A. (1985). Compared Evolution of Plasma Fibronectin and Angiotensin-converting Enzyme Levels in Septic ARDS. Chest.

[42] Reddy R., Asante I., Liu S., Parikh P., Liebler J., Borok Z., Rodgers K., Baydur A., Louie S.G. (2019). Circulating angiotensin peptides levels in Acute Respiratory Distress Syndrome correlate with clinical outcomes: A pilot study. PLoS ONE.

[43] Idell S., Kueppers F., Lippmann M., Rosen H., Niederman M., Fein A. (1987). Angiotensin Converting Enzyme in Bronchoalveolar Lavage in ARDS. Chest.

[44] Kaparianos A. (2011). Local Renin-Angiotensin II Systems, Angiotensin-Converting Enzyme and its Homologue ACE2: Their Potential Role in the Pathogenesis of Chronic Obstructive Pulmonary Diseases, Pulmonary Hypertension and Acute Respiratory Distress Syndrome. Curr. Med. Chem..

[45] Takei Y., Yamada M., Saito K., Kameyama Y., Sugiura H., Makiguchi T., Fujino N., Koarai A., Toyama H., Saito K. (2019). Increase in circulating ACE-positive endothelial microparticles during acute lung injury. Eur. Respir. J..

[46] Yamamoto T., Wang L.-M., Shimakura K., Sanaka M., Koike Y., Mineshita S. (1997). Angiotensin II-Induced Pulmonary Edema in a Rabbit Model. Jpn. J. Pharmacol..

[47] Yilin Z., Yandong N., Faguang J. (2015). Role of angiotensin-converting enzyme (ACE) and ACE2 in a rat model of smoke inhalation induced acute respiratory distress syndrome. Burns.

[48] Hermanns M.I., Müller A.M., Tsokos M., Kirkpatrick C.J. (2013). LPS-induced effects on angiotensin I-converting enzyme expression and shedding in human pulmonary microvascular endothelial cells. In Vitro Cell. Dev. Biol. Anim..

[49] Rey-Parra G.J., Vadivel A., Coltan L., Hall A., Eaton F., Schuster M., Loibner H., Penninger J.M., Kassiri Z., Oudit G.Y. (2012). Angiotensin converting enzyme 2 abrogates bleomycin-induced lung injury. J. Mol. Med..

[50] Asperen R.M.W.-V., Lutter R., Specht P.A., Moll G.N., Van Woensel J.B., Van Der Loos C.M., Van Goor H., Kamilic J., Florquin S., Bos A.P. (2011). Acute respiratory distress syndrome leads to reduced ratio of ACE/ACE2 activities and is prevented by angiotensin-(1-7) or an angiotensin II receptor antagonist. J. Pathol..

[51] Imai Y., Kuba K., Rao S., Huan Y., Guo F., Guan B., Yang P., Sarao R., Wada T., Leong-Poi H. (2005). Angiotensin-converting enzyme 2 protects from severe acute lung failure. Nat. Cell Biol..

[52] Li Y., Zeng Z., Cao Y., Liu Y., Ping F., Liang M., Xue Y., Xi C., Zhou M., Jiang W. (2016). Angiotensin-converting enzyme 2 prevents lipopolysaccharide-induced rat acute lung injury via suppressing the ERK1/2 and NF-kappaB signaling pathways. Sci. Rep..

[53] Harmer D., Gilbert M., Borman R., Clark K.L. (2002). Quantitative mRNA expression profiling of ACE 2, a novel homologue of angiotensin converting enzyme. FEBS Lett..

[54] Li M.-Y., Li L., Zhang Y., Wang X. (2020). Expression of the SARS-CoV-2 cell receptor gene ACE2 in a wide variety of human tissues. Infect. Dis. Poverty.

[55] Rigat B., Hubert C., Alhenc-Gelas F., Cambien F., Corvol P., Soubrier F. (1990). An insertion/deletion polymorphism in the angiotensin I-converting enzyme gene accounting for half the variance of serum enzyme levels. J. Clin. Investig..

[56] Marshall R.P., Webb S., Bellingan G.J., Montgomery H.E., Chaudhari B., McAnulty R.J., Humphries S.E., Hill M.R., Laurent G.J. (2002). Angiotensin Converting Enzyme Insertion/Deletion Polymorphism Is Associated with Susceptibility and Outcome in Acute Respiratory Distress Syndrome. Am. J. Respir. Crit. Care Med..

[57] Adamzik M., Frey M.U.H., Sixt S., Knemeyer L., Beiderlinden M., Peters J., Siffert W. (2007). ACE I/D but not AGT (-6)A/G polymorphism is a risk factor for mortality in ARDS. Eur. Respir. J..

[58] Matsuda A., Kishi T., Jacob A., Aziz M., Wang P. (2012). Association between insertion/deletion polymorphism in angiotensin-converting enzyme gene and acute lung injury/acute respiratory distress syndrome: A meta-analysis. BMC Med. Genet..

[59] Jerng J.-S., Yu C.-J., Wang H.-C., Chen K.-Y., Cheng S.-L., Yang P.-C. (2006). Polymorphism of the angiotensin-converting enzyme gene affects the outcome of acute respiratory distress syndrome. Crit. Care Med..

[60] Cruces P., Diaz F., Puga A., Erranz B., Donoso A., Carvajal C., Wilhelm J., Repetto G.M. (2012). Angiotensin-converting enzyme insertion/deletion polymorphism is associated with severe hypoxemia in pediatric ARDS. Intensive Care Med..

[61] Villar J., Flores C., Pérez-Méndez L., Maca-Meyer N., Espinosa E., Blanco J., Sangüesa R., Muriel A., Tejera P., Muros M. (2008). Angiotensin-converting enzyme insertion/deletion polymorphism is not associated with susceptibility and outcome in sepsis and acute respiratory distress syndrome. Intensive Care Med..

[62] Wang D., Chai X.-Q., Magnussen C.G., Zosky G.R., Shu S.-H., Wei X., Hu S.-S. (2019). Renin-angiotensin-system, a potential pharmacological candidate, in acute respiratory distress syndrome during mechanical ventilation. Pulm. Pharmacol. Ther..

[63] Zhang H., Baker A. (2017). Recombinant human ACE2: Acing out angiotensin II in ARDS therapy. Crit. Care.

[64] Kuba K., Imai Y., Rao S., Gao H., Guo F., Guan B., Huan Y., Yang P., Zhang Y., Deng W. (2005). A crucial role of angiotensin converting enzyme 2 (ACE2) in SARS coronavirus–induced lung injury. Nat. Med..

[65] Zou Z., Yan Y., Shu Y., Gao R., Sun Y., Li X., Ju X., Liang Z., Liu Q., Zhao Y. (2014). Angiotensin-converting enzyme 2 protects from lethal avian influenza A H5N1 infections. Nat. Commun..

[66] Orfanos S., Ehrhart I., Barman S., Hofman W., Catravas J. (1997). Endothelial Ectoenzyme Assays Estimate Perfused Capillary Surface Area in the Dog Lung. Microvasc. Res..

[67] Orfanos S.E., Langleben D., Khoury J., Schlesinger R.D., Dragatakis L., Roussos C., Ryan J.W., Catravas J.D. (1999). Pulmonary capillary endothelium-bound angiotensin-converting enzyme activity in humans. Circulation.

[68] Kaziani K., Vassiliou A., Kotanidou A., Athanasiou C., Korovesi I., Glynos K., Orfanos S.E. (2018). Activated Protein C has No Effect on Pulmonary Capillary Endothelial Function in Septic Patients with Acute Respiratory Distress Syndrome: Association of Endothelial Dysfunction with Mortality. Infect. Dis. Ther..

[69] Langleben D., Orfanos S.E., Giovinazzo M., Schlesinger R.D., Hirsch A.M., Blenkhorn F., Lesenko L., Armaganidis A., Catravas J.D. (2015). Acute Vasodilator Responsiveness and Microvascular Recruitment in Idiopathic Pulmonary Arterial Hypertension. Ann. Intern. Med..

[70] Glynos C., Athanasiou C., Kotanidou A., Korovesi I., Kaziani K., Livaditi O., Dimopoulou I., Maniatis N.A., Tsangaris I., Roussos C. (2013). Preclinical Pulmonary Capillary Endothelial Dysfunction is Present in Brain Dead Subjects. Pulm. Circ..

[71] Orfanos S.E., Armaganidis A., Glynos C., Psevdi E., Kaltsas P., Sarafidou P., Catravas J.D., Dafni U.G., Langleben D., Roussos C. (2000). Pulmonary Capillary Endothelium-Bound Angiotensin-Converting Enzyme Activity in Acute Lung Injury. Circulation.

[72] Orfanos S.E., Hirsch A.M., Giovinazzo M., Armaganidis A., Catravas J.D., Langleben D. (2008). Pulmonary capillary endothelial metabolic function in chronic thromboembolic pulmonary hypertension. J. Thromb. Haemost..

[73] Orfanos S.E., Psevdi E., Stratigis N., Langleben D., Catravas J.D., Kyriakidis M., Moutsopoulos H.M., Roussos C., Vlachoyiannopoulos P.G. (2001). Pulmonary capillary endothelial dysfunction in early systemic sclerosis. Arthritis Rheum..

[74] Langleben D., Orfanos S.E., Giovinazzo M., Hirsch A., Baron M., Armaganidis A., Catravas J.D., Senécal J.-L. (2008). Pulmonary capillary endothelial metabolic dysfunction: Severity in pulmonary arterial hypertension related to connective tissue disease versus idiopathic pulmonary arterial hypertension. Arthritis Rheum..

[75] Dudzinski D.M., Igarashi J., Greif D., Michel T. (2006). The regulation and pharmacology of endothelial nitric oxide synthase. Annu. Rev. Pharmacol. Toxicol..

[76] Abdih H., Kelly C.J., Bouchier-Hayes D.J., Watson R.W., Redmond H., Burke P., Bouchier-Hayes D.J., William R. (1994). Nitric Oxide (Endothelium-Derived Relaxing Factor) Attenuates Revascularization-Induced Lung Injury. J. Surg. Res..

[77] Garrean S., Gao X., Brovkovych V., Shimizu J., Zhao Y.-Y., Vogel S.M., Malik A.B. (2006). Caveolin-1 regulates NF-kappaB activation and lung inflammatory response to sepsis induced by lipopolysaccharide. J. Immunol..

[78] Kaminski A., Pohl C.B., Sponholz C., Ma N., Stamm C., Vollmar B., Steinhoff G. (2004). Up-Regulation of Endothelial Nitric Oxide Synthase Inhibits Pulmonary Leukocyte Migration Following Lung Ischemia-Reperfusion in Mice. Am. J. Pathol..

[79] Kaminski A., Kasch C., Zhang L., Kumar S., Sponholz C., Choi Y.-H., Ma N., Liebold A., Ladilov Y., Steinhoff G. (2007). Endothelial nitric oxide synthase mediates protective effects of hypoxic preconditioning in lungs. Respir. Physiol. Neurobiol..

[80] Takenaka K., Nishimura Y., Nishiuma T., Sakashita A., Yamashita T., Kobayashi K., Satouchi M., Ishida T., Kawashima S., Yokoyama M. (2006). Ventilator-induced lung injury is reduced in transgenic mice that overexpress endothelial nitric oxide synthase. Am. J. Physiol. Cell. Mol. Physiol..

[81] Yamashita T., Kawashima S., Ohashi Y., Ozaki M., Ueyama T., Ishida T., Inoue N., Hirata K.-I., Akita H., Yokoyama M. (2000). Resistance to endotoxin shock in transgenic mice overexpressing endothelial nitric oxide synthase. Circulation.

[82] Zhang L., Kumar S., Kaminski A., Kasch C., Sponholz C., Stamm C., Ladilov Y., Steinhoff G. (2006). Importance of endothelial nitric oxide synthase for the hypothermic protection of lungs against ischemia-reperfusion injury. J. Thorac. Cardiovasc. Surg..

[83] Gielis J.F., Quirynen L., Briedé J.J., Roelant E., Cos P., Van Schil P.E. (2017). Pathogenetic role of endothelial nitric oxide synthase uncoupling during lung ischaemia–reperfusion injury. Eur. J. Cardio-Thoracic Surg..

[84] Drucker N.A., Jensen A.R., Winkel J.P.T., Ferkowicz M.J., Markel T.A. (2018). Loss of endothelial nitric oxide synthase exacerbates intestinal and lung injury in experimental necrotizing enterocolitis. J. Pediatr. Surg..

[85] Forstermann U., Munzel T. (2006). Endothelial Nitric Oxide Synthase in Vascular Disease. Circulation.

[86] Gow A.J., Thom S.R., Ischiropoulos H. (1998). Nitric oxide and peroxynitrite-mediated pulmonary cell death. Am. J. Physiol. Content.

[87] Weinberger B., Djerad A., Monier C., Houzé P., Borron S.W., Lefauconnier J.-M., Baud F.J. (2001). The Toxicology of Inhaled Nitric Oxide. Toxicol. Sci..

[88] Kristof A.S., Goldberg P., Laubach V., Hussain S.N.A. (1998). Role of Inducible Nitric Oxide Synthase in Endotoxin-induced Acute Lung Injury. Am. J. Respir. Crit. Care Med..

[89] Gross C.M., Rafikov R., Kumar S., Aggarwal S., Iii P.B.H., Meadows M.L., Cherian-Shaw M., Kangath A., Sridhar S., Lucas R. (2015). Endothelial Nitric Oxide Synthase Deficient Mice Are Protected from Lipopolysaccharide Induced Acute Lung Injury. PLoS ONE.

[90] Song J., Palmer K., Sun B. (2010). Effects of inhaled nitric oxide and surfactant with extracorporeal life support in recovery phase of septic acute lung injury in piglets. Pulm. Pharmacol. Ther..

[91] Hart C.M. (1999). Nitric Oxide in Adult Lung Disease. Chest.

[92] Zayek M., Wild L., Roberts J.D., Morin F.C. (1993). Effect of nitric oxide on the survival rate and incidence of lung injury in newborn lambs with persistent pulmonary hypertension. J. Pediatr..

[93] Rossaint R., Falke K., Lopez F., Slama K., Pison U., Zapol W.M. (1993). Inhaled Nitric Oxide for the Adult Respiratory Distress Syndrome. N. Engl. J. Med..

[94] Benzing A., Geiger K. (1994). Inhaled nitric oxide lowers pulmonary capillary pressure and changes longitudinal distribution of pulmonary vascular resistance in patients with acute lung injury. Acta Anaesthesiol. Scand..

[95] Michael J.R., Barton R.G., Saffle J.R., Mone M., Markewitz B.A., Hillier K., Elstad M.R., Campbell E.J., Troyer B.E., Whatley R.E. (1998). Inhaled Nitric Oxide Versus Conventional Therapy. Am. J. Respir. Crit. Care Med..

[96] Troncy E., Collet J.-P., Shapiro S., Guimond J.-G., Blair L., Ducruet T., Francoeur M., Charbonneau M., Blaise G. (1998). Inhaled Nitric Oxide in Acute Respiratory Distress Syndrome. Am. J. Respir. Crit. Care Med..

[97] Dellinger R.P., Zimmerman J.L., Taylor R.W., Straube R.C., Hauser D.L., Criner G.J., Davis K., Hyers T.M., Papadakos P. (1998). Effects of inhaled nitric oxide in patients with acute respiratory distress syndrome. Crit. Care Med..

[98] Lundin S., Mang H., Smithies M., Stenqvist O., Frostell C. (1999). Inhalation of nitric oxide in acute lung injury: Results of a European multicentre study. Intensive Care Med..

[99] Taylor R.W., Zimmerman J.L., Dellinger R.P., Straube R.C., Criner G.J., Davis J.K., Kelly K.M., Smith T.C., Small R.J. (2004). Low-Dose Inhaled Nitric Oxide in Patients with Acute Lung InjuryA Randomized Controlled Trial. JAMA.

[100] Adhikari N.K.J., Burns K.E., Friedrich J.O., Granton J.T., Cook D.J., Meade M.O. (2007). Effect of nitric oxide on oxygenation and mortality in acute lung injury: Systematic review and meta-analysis. BMJ.

[101] Afshari A., Brok J., Moller A.M., Wetterslev J. (2010). Inhaled nitric oxide for acute respiratory distress syndrome (ARDS) and acute lung injury in children and adults. Cochrane Database Syst. Rev..

[102] Adhikari N.K.J., Dellinger R.P., Lundin S., Payen D., Vallet B., Gerlach H., Park K.J., Mehta S., Slutsky A.S., Friedrich J.O. (2014). Inhaled Nitric Oxide Does Not Reduce Mortality in Patients with Acute Respiratory Distress Syndrome Regardless of Severity. Crit. Care Med..

[103] Gebistorf F., Karam O., Wetterslev J., Afshari A. (2016). Inhaled nitric oxide for acute respiratory distress syndrome (ARDS) in children and adults. Cochrane Database Syst. Rev..

[104] Ruan S.-Y., Huang T.-M., Wu H.-Y., Wu H.-D., Jann-Yuan W., Lai M.-S. (2015). Inhaled nitric oxide therapy and risk of renal dysfunction: A systematic review and meta-analysis of randomized trials. Crit. Care.

[105] Ruan S.-Y., Wu H.-Y., Lin H.-H., Wu H.-D., Yu C.-J., Lai M.-S. (2016). Inhaled nitric oxide and the risk of renal dysfunction in patients with acute respiratory distress syndrome: A propensity-matched cohort study. Crit. Care.

[106] Mitchell J.A., Ali F., Bailey L., Moreno L., Harrington L.S. (2008). Role of nitric oxide and prostacyclin as vasoactive hormones released by the endothelium. Exp. Physiol..

[107] Walmrath D., Schneider T., Pilch J., Grimminger F., Seeger W. (1993). Aerosolised prostacyclin in adult respiratory distress syndrome. Lancet.

[108] Zwissler B., Kemming G., Habler O., Kleen M., Merkel M., Haller M., Briegel J., Welte M., Peter K. (1996). Inhaled prostacyclin (PGI2) versus inhaled nitric oxide in adult respiratory distress syndrome. Am. J. Respir. Crit. Care Med..

[109] Kallet R.H., Burns G., Zhuo H., Ho K., Phillips J.S., Pangilinan L.P., Yip V., Gomez A., Lipnick M.S. (2017). Severity of Hypoxemia and Other Factors That Influence the Response to Aerosolized Prostacyclin in ARDS. Respir. Care.

[110] Searcy R.J., Morales J.R., Ferreira J., Johnson D.W. (2015). The role of inhaled prostacyclin in treating acute respiratory distress syndrome. Ther. Adv. Respir. Dis..

[111] Afshari A., Bille A.B., Allingstrup M. (2017). Aerosolized prostacyclins for acute respiratory distress syndrome (ARDS). Cochrane Database Syst. Rev..

[112] Levi M., Cate H.T., Van Der Poll T. (2002). Endothelium: Interface between coagulation and inflammation. Crit. Care Med..

[113] Mantovani A., Bussolino F., Introna M. (1997). Cytokine regulation of endothelial cell function: From molecular level to the bedside. Immunol. Today.

[114] Muller W.A. (2014). How Endothelial Cells Regulate Transmigration of Leukocytes in the Inflammatory Response. Am. J. Pathol..

[115] Levi M., Schultz M. (2008). The inflammation-coagulation axis as an important intermediate pathway in acute lung injury. Crit. Care.

[116] Meduri G.U., Kohler G., Headley S., Tolley E., Stentz F., Postlethwaite A. (1995). Inflammatory Cytokines in the BAL of Patients With ARDS. Chest.

[117] Park W.Y., Goodman R.B., Steinberg K.P., Ruzinski J.T., Radella F., Park D.R., Pugin J., Skerrett S.J., Hudson L.D., Martin T.R. (2001). Cytokine Balance in the Lungs of Patients with Acute Respiratory Distress Syndrome. Am. J. Respir. Crit. Care Med..

[118] Meduri G.U., Headley S., Kohler G., Stentz F., Tolley E., Umberger R., Leeper K. (1995). Persistent elevation of inflammatory cytokines predicts a poor outcome in ARDS. Plasma IL-1 beta and IL-6 levels are consistent and efficient predictors of outcome over time. Chest.

[119] Cepkova M., Brady S., Sapru A., Matthay M.A., Church G.D. (2006). Biological markers of lung injury before and after the institution of positive pressure ventilation in patients with acute lung injury. Crit. Care.

[120] Nakamura T., Sato E., Fujiwara N., Kawagoe Y., Maeda S., Yamagishi S.-I. (2011). Increased levels of soluble receptor for advanced glycation end products (sRAGE) and high mobility group box 1 (HMGB1) are associated with death in patients with acute respiratory distress syndrome. Clin. Biochem..

[121] Swaroopa D., Bhaskar K., Mahathi T., Katkam S., Raju Y.S., Chandra N., Kutala V.K. (2016). Association of serum interleukin-6, interleukin-8, and Acute Physiology and Chronic Health Evaluation II score with clinical outcome in patients with acute respiratory distress syndrome. Indian J. Crit. Care Med..

[122] Donnelly T.J., Meade P., Jagels M., Cryer H.G., Law M.M., Hugli T.E., Shoemaker W.C., Abraham E. (1994). Cytokine, complement, and endotoxin profiles associated with the development of the adult respiratory distress syndrome after severe injury. Crit. Care Med..

[123] Meade P., Shoemaker W.C., Donnelly T.J., Abraham E., Jagels M.A., Cryer H.G., Hugli T.E., Bishop M.H., Wo C.C.J. (1994). Temporal patterns of hemodynamics, oxygen transport, cytokine activity, and complement activity in the development of adult respiratory distress syndrome after severe injury. J. Trauma Inj. Infect. Crit. Care.

[124] Stüber F., Wrigge H., Schroeder S., Wetegrove S., Zinserling J., Hoeft A., Putensen C. (2002). Kinetic and reversibility of mechanical ventilation-associated pulmonary and systemic inflammatory response in patients with acute lung injury. Intensive Care Med..

[125] Ranieri V.M., Suter P.M., Tortorella C., De Tullio R., Dayer J.M., Brienza A., Bruno F., Slutsky A.S. (1999). Effect of Mechanical Ventilation on Inflammatory Mediators in Patients with Acute Respiratory Distress Syndrome. JAMA.

[126] Voiriot G., Razazi K., Amsellem V., Van Nhieu J.T., Abid S., Adnot S., Dessap A.M., Maitre B. (2017). Interleukin-6 displays lung anti-inflammatory properties and exerts protective hemodynamic effects in a double-hit murine acute lung injury. Respir. Res..

[127] Qin M., Qiu Z. (2019). Changes in TNF-α, IL-6, IL-10 and VEGF in rats with ARDS and the effects of dexamethasone. Exp. Ther. Med..

[128] Donnelly S.C., Haslett C., Strieter R.M., Kunkel S.L., Walz A., Robertson C.R., Carter D.C., Pollok A.J., Grant I.S. (1993). Interleukin-8 and development of adult respiratory distress syndrome in at-risk patient groups. Lancet.

[129] Calfee C.S., Ware L.B., Glidden D.V., Eisner M.D., Parsons P.E., Thompson B.T., Matthay M.A. (2011). Use of risk reclassification with multiple biomarkers improves mortality prediction in acute lung injury. Crit. Care Med..

[130] McClintock D., Zhuo H., Wickersham N., Matthay M.A., Ware L.B. (2008). Biomarkers of inflammation, coagulation and fibrinolysis predict mortality in acute lung injury. Crit. Care.

[131] Ware L.B., Koyama T., Billheimer D.D., Wu W., Bernard G.R., Thompson B.T., Brower R.G., Standiford T.J., Martin T.R., Matthay M.A. (2010). Prognostic and Pathogenetic Value of Combining Clinical and Biochemical Indices in Patients with Acute Lung Injury. Chest.

[132] Flori H.R., Sapru A., Quasney M.W., Gildengorin G., Curley M.A., Matthay M.A., Dahmer M.K. (2019). A prospective investigation of interleukin-8 levels in pediatric acute respiratory failure and acute respiratory distress syndrome. Crit. Care.

[133] Laffon M., Pittet J.-F., Modelska K., Matthay M.A., Young D.M. (1999). Interleukin-8 Mediates Injury from Smoke Inhalation to both the Lung Endothelial and the Alveolar Epithelial Barriers in Rabbits. Am. J. Respir. Crit. Care Med..

[134] Osman M.O., Kristensen J.U., Jacobsen N.O., Lausten S.B., Deleuran B., Gesser B., Matsushima K., Larsen C.G., Jensen S.L. (1998). A monoclonal anti-interleukin 8 antibody (WS-4) inhibits cytokine response and acute lung injury in experimental severe acute necrotising pancreatitis in rabbits. Gut.

[135] Sekido N., Mukaida N., Harada A., Nakanishi I., Watanabe Y., Matsushima K. (1993). Prevention of lung reperfusion injury in rabbits by a monoclonal antibody against interleukin-8. Nat. Cell Biol..

[136] Bao Z., Ye Q., Gong W., Xiang Y., Wan H. (2010). Humanized monoclonal antibody against the chemokine CXCL-8 (IL-8) effectively prevents acute lung injury. Int. Immunopharmacol..

[137] Hoegl S., Boost K.A., Czerwonka H., Dolfen A., Scheiermann P., Mühl H., Zwissler B., Hofstetter C. (2009). Inhaled IL-10 reduces biotrauma and mortality in a model of ventilator-induced lung injury. Respir. Med..

[138] Wu J., Xiong Z., Xiong G., Ding F., Lei J., Lu S., Li Y., He G., Zhao L., Liu Z. (2015). Protective effect of interleukin-10 and recombinant human keratinocyte growth factor-2 on ventilation-induced lung injury in rats. Genet. Mol. Res..

[139] Chen J., Lin J., Luo H., Li M. (2019). Effects of Human Interleukin-10 on Ventilator-Associated Lung Injury in Rats. Inflammation.

[140] Aisiku I.P., Yamal J.-M., Doshi P., Benoit J.S., Gopinath S., Goodman J.C., Robertson C.S. (2016). Plasma cytokines IL-6, IL-8, and IL-10 are associated with the development of acute respiratory distress syndrome in patients with severe traumatic brain injury. Crit. Care.

[141] Belopolskaya O.B., Smelaya T.V., Moroz V.V., Golubev A.M., Salnikova L.E. (2015). Clinical associations of host genetic variations in the genes of cytokines in critically ill patients. Clin. Exp. Immunol..

[142] Abraham E. (2003). Neutrophils and acute lung injury. Crit. Care Med..

[143] Rebetz J., Semple J.W., Kapur R. (2018). The Pathogenic Involvement of Neutrophils in Acute Respiratory Distress Syndrome and Transfusion-Related Acute Lung Injury. Transfus. Med. Hemother..

[144] Grunwell J.R., Stephenson S.T., Mohammad A.F., Jones K., Mason C., Opolka C., Fitzpatrick A.M. (2020). Differential type I interferon response and primary airway neutrophil extracellular trap release in children with acute respiratory distress syndrome. Sci. Rep..

[145] Zhao J., Zhang G., Cui W., Tian B. (2020). Progress of neutrophil extracellular traps in airway inflammation of acute lung injury/acute respiratory distress syndrome: Review. Chin. J. Cell. Mol. Immunol..

[146] Middleton E.A., He X.-Y., Denorme F., Campbell R.A., Ng D., Salvatore S.P., Mostyka M., Baxter-Stoltzfus A., Borczuk A.C., Loda M. (2020). Neutrophil extracellular traps contribute to immunothrombosis in COVID-19 acute respiratory distress syndrome. Blood.

[147] Yildiz C., Palaniyar N., Otulakowski G., Khan M.A., Post M., Kuebler W.M., Tanswell K., Belcastro R., Masood A., Engelberts D. (2015). Mechanical Ventilation Induces Neutrophil Extracellular Trap Formation. Anesthesiology.

[148] Tedder T.F., Steeber D.A., Chen A., Engel P. (1995). The selecting: Vascular adhesion molecules. FASEB J..

[149] Patel K.D., Cuvelier S.L., Wiehlera S. (2002). Selectins: Critical mediators of leukocyte recruitment. Semin. Immunol..

[150] Donnelly S.C., Haslett C., Dransfield I., Robertson C.E., Carter D.C., Ross J.A., Grant I.S., Tedder T.F. (1994). Role of selectins in development of adult respiratory distress syndrome. Lancet.

[151] Sakamaki F., Ishizaka A., Handa M., Fujishima S., Urano T., Sayama K., Nakamura H., Kanazawa M., Kawashiro T., Katayama M. (1995). Soluble form of P-selectin in plasma is elevated in acute lung injury. Am. J. Respir. Crit. Care Med..

[152] Xu Z., Wu G.-M., Li Q., Ji F.-Y., Shi Z., Guo H., Yin J.-B., Zhou J., Gong L., Mei C.-X. (2018). Predictive Value of Combined LIPS and ANG-2 Level in Critically Ill Patients with ARDS Risk Factors. Mediat. Inflamm..

[153] Okajima K., Harada N., Sakurai G., Soga Y., Suga H., Terada T., Nakagawa T. (2006). Rapid assay for plasma soluble E-selectin predicts the development of acute respiratory distress syndrome in patients with systemic inflammatory response syndrome. Transl. Res..

[154] Burnham E.L., Moss M., Harris F., Brown L.A.S. (2004). Elevated plasma and lung endothelial selectin levels in patients with acute respiratory distress syndrome and a history of chronic alcohol abuse. Crit. Care Med..

[155] Ruchaud-Sparagano M.-H., Drost E.M., Donnelly S.C., Bird M.I., Haslett C., Dransfield I. (1998). Potential pro-inflammatory effects of soluble E-selectin upon neutrophil function. Eur. J. Immunol..

[156] Al-Biltagi M., Abo-Elezz A.A.E., Elshafiey R.M.G., Suliman G.A., Mabrouk M.M., Mourad H.A. (2016). The predictive value of soluble endothelial selectin plasma levels in children with acute lung injury. J. Crit. Care.

[157] Carraway M.S., Welty-Wolf K.E., Kantrow S.P., Huang Y.-C.T., Simonson S.G., Que L.G., Kishimoto T.K., Piantadosi C.A. (1998). Antibody to E- and L-Selectin Does Not Prevent Lung Injury or Mortality in Septic Baboons. Am. J. Respir. Crit. Care Med..

[158] Ridings P.C., Windsor A.C.J., Jutila M.A., Blocher C.R., Fisher B.J., Sholley M.M., Sugerman H.J., Fowler A.A. (1995). A dual-binding antibody to E- and L-selectin attenuates sepsis-induced lung injury. Am. J. Respir. Crit. Care Med..

[159] Chandra A., Katahira J., Schmalstieg F.C., Murakami K., Enkhbaatar P., Cox R.A., Hawkins H.K., Traber L.D., Herndon D.N., Traber D.L. (2003). P-selectin blockade fails to improve acute lung injury in sheep. Clin. Sci..

[160] Doerschuk C.M., Quinlan W.M., Doyle N.A., Bullard D.C., Vestweber D., Jones M.L., Takei F., Ward P.A., Beaudet A.L. (1996). The role of P-selectin and ICAM-1 in acute lung injury as determined using blocking antibodies and mutant mice. J. Immunol..

[161] Hayashi H., Koike H., Kurata Y., Imanishi N., Tojo S.J. (1999). Protective effects of sialyl Lewis X and anti-P-selectin antibody against lipopolysaccharide-induced acute lung injury in rabbits. Eur. J. Pharmacol..

[162] Zarbock A., Singbartl K., Ley K. (2006). Complete reversal of acid-induced acute lung injury by blocking of platelet-neutrophil aggregation. J. Clin. Investig..

[163] Bime C., Pouladi N., Sammani S., Batai K., Casanova N., Zhou T., Kempf C.L., Sun X., Camp S.M., Wang T. (2018). Genome-Wide Association Study in African Americans with Acute Respiratory Distress Syndrome Identifies the Selectin P Ligand Gene as a Risk Factor. Am. J. Respir. Crit. Care Med..

[164] Reutershan J., Ley K. (2004). Bench-to-bedside review: Acute respiratory distress syndrome—How neutrophils migrate into the lung. Crit. Care.

[165] Moss M., Gillespie M.K., Ackerson L., Moore F.A., Moore E.E., Parsons P.E. (1996). Endothelial cell activity varies in patients at risk for the adult respiratory distress syndrome. Crit. Care Med..

[166] Skiba-Choińska I., Rogowski F. (1996). Adhesion molecules and their role in pathogenesis of ARDS. Prz. Lek..

[167] Kimura D., Saravia J., Rovnaghi C.R., Meduri G.U., Schwingshackl A., Cormier S.A., Anand K.J. (2016). Plasma Biomarker Analysis in Pediatric ARDS: Generating Future Framework from a Pilot Randomized Control Trial of Methylprednisolone: A Framework for Identifying Plasma Biomarkers Related to Clinical Outcomes in Pediatric ARDS. Front. Pediatr..

[168] Müller A.M., Cronen C., Müller K.-M., Kirkpatrick C.J. (2002). Heterogeneous expression of cell adhesion molecules by endothelial cells in ARDS. J. Pathol..

[169] Reiss L.K., Uhlig U., Uhlig S. (2012). Models and mechanisms of acute lung injury caused by direct insults. Eur. J. Cell Biol..

[170] Bedirli A., Kerem M., Pasaoglu H., Akyurek N., Tezcaner T., Elbeg S., Memis L., Sakrak O. (2007). Beta-glucan attenuates inflammatory cytokine release and prevents acute lung injury in an experimental model of sepsis. Shock.

[171] Chen L., Li W., Qi D., Lu L., Zhang Z., Wang D. (2018). Honokiol protects pulmonary microvascular endothelial barrier against lipopolysaccharide-induced ARDS partially via the Sirt3/AMPK signaling axis. Life Sci..

[172] He B., Geng S., Zhou W., Rui Y., Mu X., Zhang C., You Q., Su X. (2018). MMI-0100 ameliorates lung inflammation in a mouse model of acute respiratory distress syndrome by reducing endothelial expression of ICAM-1. Drug Des. Dev. Ther..

[173] Lang S., Li L., Wang X., Sun J., Xue X., Xiao Y., Zhang M., Ao T., Wang J. (2017). CXCL10/IP-10 Neutralization Can Ameliorate Lipopolysaccharide-Induced Acute Respiratory Distress Syndrome in Rats. PLoS ONE.

[174] Félétou M., Vanhoutte P.M. (2006). Endothelial dysfunction: A multifaceted disorder (The Wiggers Award Lecture). Am. J. Physiol. Circ. Physiol..

[175] Edelberg J.M., Christie P.D., Rosenberg R.D. (2001). Regulation of Vascular Bed–Specific Prothrombotic Potential. Circ. Res..

[176] Sabharwal A.K., Bajaj S.P., Ameri A., Tricomi S.M., Hyers T.M., Dahms T.E., Taylor F.B., Bajaj M.S. (1995). Tissue factor pathway inhibitor and von Willebrand factor antigen levels in adult respiratory distress syndrome and in a primate model of sepsis. Am. J. Respir. Crit. Care Med..

[177] Scarpati E.M., Sadler J.E. (1989). Regulation of endothelial cell coagulant properties. Modulation of tissue factor, plasminogen activator inhibitors, and thrombomodulin by phorbol 12-myristate 13-acetate and tumor necrosis factor. J. Biol. Chem..

[178] Siemiatkowski A., Kłoczko J., Galar M., Czaban S.L. (2000). Von Willebrand Factor Antigen as a Prognostic Marker in Posttraumatic Acute Lung Injury. Pathophysiol. Haemost. Thromb..

[179] Ware L.B., Eisner M.D., Thompson B.T., Parsons P.E., Matthay M.A. (2004). Significance of Von Willebrand Factor in Septic and Nonseptic Patients with Acute Lung Injury. Am. J. Respir. Crit. Care Med..

[180] Hou P.C., Filbin M.R., Wang H., Ngo L., Huang D.T., Aird W.C., Yealy D.M., Angus D.C., Kellum J.A., Shapiro N.I. (2017). Endothelial Permeability and Hemostasis in Septic Shock. Chest.

[181] Afshar M., Burnham E.L., Joyce C., Gagnon R., Dunn R., Albright J.M., Ramirez L., Repine J.E., Netzer G., Kovacs E.J. (2019). Injury Characteristics and von Willebrand Factor for the Prediction of Acute Respiratory Distress Syndrome in Patients With Burn Injury: Development and Internal Validation. Ann. Surg..

[182] Rubin D.B., Wiener-Kronish J.P., Murray J.F., Green D.R., Turner J., Luce J.M., Montgomery A.B., Marks J.D., Matthay M.A. (1990). Elevated von Willebrand factor antigen is an early plasma predictor of acute lung injury in nonpulmonary sepsis syndrome. J. Clin. Investig..

[183] El Basset Abo El Ezz A.A., Abd El Hafez M.A., El Amrousy D.M., El Momen Suliman G.A. (2017). The predictive value of Von Willebrand factor antigen plasma levels in children with acute lung injury. Pediatr. Pulmonol..

[184] Moss M., Ackerson L., Gillespie M.K., Moore F.A., Moore E.E., Parsons P.E. (1995). Von Willebrand factor antigen levels are not predictive for the adult respiratory distress syndrome. Am. J. Respir. Crit. Care Med..

[185] Bajaj M.S., Tricomi S.M. (1999). Plasma levels of the three endothelial-specific proteins von Willebrand factor, tissue factor pathway inhibitor, and thrombomodulin do not predict the development of acute respiratory distress syndrome. Intensive Care Med..

[186] Agrawal A., Matthay M.A., Kangelaris K.N., Stein J., Chu J.C., Imp B.M., Cortez A., Abbott J., Liu K.D., Calfee C.S. (2013). Plasma Angiopoietin-2 Predicts the Onset of Acute Lung Injury in Critically Ill Patients. Am. J. Respir. Crit. Care Med..

[187] Hendrickson C.M., Matthay M.A. (2018). Endothelial biomarkers in human sepsis: Pathogenesis and prognosis for ARDS. Pulm. Circ..

[188] Frantzeskaki F., Armaganidis A., Orfanos S.E. (2017). Immunothrombosis in Acute Respiratory Distress Syndrome: Cross Talks between Inflammation and Coagulation. Respiration.

[189] Yau J.W., Teoh H., Verma S. (2015). Endothelial cell control of thrombosis. BMC Cardiovasc. Disord..

[190] Monroe D.M., Key N.S. (2007). The tissue factor-factor VIIa complex: Procoagulant activity, regulation, and multitasking. J. Thromb. Haemost..

[191] Esmon C.T. (2003). The Protein C Pathway. Chest.

[192] Vassiliou A.G., Kotanidou A., Mastora Z., Maniatis N.A., Albani P., Jahaj E., Koutsoukou A., Armaganidis A., Orfanos S.E. (2015). Elevated soluble endothelial protein C receptor levels at ICU admission are associated with sepsis development. Minerva Anestesiol..

[193] Block E.R. (1992). Pulmonary Endothelial Cell Pathobiology: Implications for Acute Lung Injury. Am. J. Med Sci..

[194] Millar F.R., Summers C., Griffiths M.J., Toshner M.R., Proudfoot A.G. (2016). The pulmonary endothelium in acute respiratory distress syndrome: Insights and therapeutic opportunities. Thorax.

[195] Horn S., Lang S., Fukudome K., Nahrup A.S., Hoffmann U., Kaden J.J., Borggrefe M., Haase K.K., Brueckmann M., Huhle G. (2005). Recombinant human activated protein C upregulates cyclooxygenase-2 expression in endothelial cells via binding to endothelial cell protein C receptor and activation of protease-activated receptor-1. Thromb. Haemost..

[196] Mosnier L.O., Yang X.V., Griffin J.H. (2007). Activated Protein C Mutant with Minimal Anticoagulant Activity, Normal Cytoprotective Activity, and Preservation of Thrombin Activable Fibrinolysis Inhibitor-dependent Cytoprotective Functions. J. Biol. Chem..

[197] Gando S., Nanzaki S., Morimoto Y., Kobayashi S., Kemmotsu O. (1999). Systemic Activation of Tissue-Factor Dependent Coagulation Pathway in Evolving Acute Respiratory Distress Syndrome in Patients with Trauma and Sepsis. J. Trauma Inj. Infect. Crit. Care.

[198] Carraway M., Ortel T., Piantadosi C., Welty-Wolf K.E. (2002). Coagulation and Inflammation in Acute Lung Injury. Thromb. Haemost..

[199] Van Der Poll T. (2008). Tissue factor as an initiator of coagulation and inflammation in the lung. Crit. Care.

[200] Idell S., Gonzalez K., Bradford H., MacArthur C.K., Fein A.M., Maunder R.J., Garcia J.G., Griffith D.E., Weiland J., Martin T.R. (1987). Procoagulant activity in bronchoalveolar lavage in the adult respiratory distress syndrome. Contribution of tissue factor associated with factor VII. Am. Rev. Respir. Dis..

[201] Ware L.B., Fang X., Matthay M.A. (2003). Protein C and thrombomodulin in human acute lung injury. Am. J. Physiol. Cell. Mol. Physiol..

[202] MacGregor I.R., Perrie A.M., Donnelly S.C., Haslett C. (1997). Modulation of human endothelial thrombomodulin by neutrophils and their release products. Am. J. Respir. Crit. Care Med..

[203] Gando S., Kameue T., Matsuda N., Sawamura A., Hayakawa M., Kato H. (2004). Systemic Inflammation and Disseminated Intravascular Coagulation in Early Stage of ALI and ARDS: Role of Neutrophil and Endothelial Activation. Inflammation.

[204] Distefano G., Romeo M.G., Betta P., Rodono’ A., Amato M. (1998). Thrombomodulin serum levels in ventilated preterm babies with respiratory distress syndrome. Eur. J. Nucl. Med. Mol. Imaging.

[205] Orwoll B., Spicer A.C., Zinter M.S., Alkhouli M.F., Khemani R.G., Flori H.R., Neuhaus J., Calfee C.S., Matthay M.A., Sapru M.A. (2015). Elevated soluble thrombomodulin is associated with organ failure and mortality in children with acute respiratory distress syndrome (ARDS): A prospective observational cohort study. Crit. Care.

[206] Suzuki A., Taniguchi H., Kondoh Y., Ando M., Watanabe N., Kimura T., Kataoka K., Yokoyama T., Sakamoto K., Hasegawa Y. (2017). Soluble thrombomodulin in bronchoalveolar lavage fluid is an independent predictor of severe drug-induced lung injury. Respirology.

[207] Sapru A., Network T.N.A., Calfee C.S., Liu K.D., Kangelaris K., Hansen H., Pawlikowska L., Ware L.B., Alkhouli M.F., Abbott J. (2015). Plasma soluble thrombomodulin levels are associated with mortality in the acute respiratory distress syndrome. Intensive Care Med..

[208] Ware L.B., Matthay M.A., Parsons P.E., Thompson B.T., Januzzi J.L., Eisner M.D. (2007). Pathogenetic and prognostic significance of altered coagulation and fibrinolysis in acute lung injury/acute respiratory distress syndrome. Crit. Care Med..

[209] Ortolan L.S., Sercundes M.K., Moura G.C., Quirino T.D.C., Debone D., Costa D.D.S., Murillo O., Marinho C.R.F., Epiphanio S. (2019). Endothelial Protein C Receptor Could Contribute to Experimental Malaria-Associated Acute Respiratory Distress Syndrome. J. Immunol. Res..

[210] Maknitikul S., Luplertlop N., Grau G.E.R., Ampawong S. (2017). Dysregulation of pulmonary endothelial protein C receptor and thrombomodulin in severe falciparum malaria-associated ARDS relevant to hemozoin. PLoS ONE.

[211] Sapru A., Network T.N.A., Liu K.D., Wiemels J., Hansen H., Pawlikowska L., Poon A., Jorgenson E., Witte J.S., Calfee C.S. (2016). Association of common genetic variation in the protein C pathway genes with clinical outcomes in acute respiratory distress syndrome. Crit. Care.

[212] Ware L.B., Bastarache J.A., Wang L. (2005). Coagulation and fibrinolysis in human acute lung injury-New therapeutic targets?. Keio J. Med..

[213] Whyte C.S., Morrow G.B., Mitchell J.L., Chowdary P., Mutch N.J. (2020). Fibrinolytic abnormalities in acute respiratory distress syndrome (ARDS) and versatility of thrombolytic drugs to treat COVID-19. J. Thromb. Haemost..

[214] Grau G.E., De Moerloose P., Bulla O., Lou J., Lei Z., Reber G., Mili N., Ricou B., Morel D.R., Suter P.M. (1997). Haemostatic Properties of Human Pulmonary and Cerebral Microvascular Endothelial Cells. Thromb. Haemost..

[215] Ozolina A., Sarkele M., Sabelnikovs O., Skesters A., Jaunalksne I., Serova J., Ievins T., Bjertnaes L.J., Vanags I. (2016). Activation of Coagulation and Fibrinolysis in Acute Respiratory Distress Syndrome: A Prospective Pilot Study. Front. Med..

[216] El Solh A.A., Bhora M., Pineda L., Aquilina A., Abbetessa L., Berbary E. (2006). Alveolar plasminogen activator inhibitor-1 predicts ARDS in aspiration pneumonitis. Intensive Care Med..

[217] Prabhakaran P., Ware L.B., White K.E., Cross M.T., Matthay M.A., Olman M.A. (2003). Elevated levels of plasminogen activator inhibitor-1 in pulmonary edema fluid are associated with mortality in acute lung injury. Am. J. Physiol. Cell. Mol. Physiol..

[218] Günther A., Mosavi P., Heinemann S., Ruppert C., Muth H., Markart P., Grimminger F., Walmrath D., Temmesfeld-Wollbrück B., Seeger W. (2000). Alveolar Fibrin Formation Caused by Enhanced Procoagulant and Depressed Fibrinolytic Capacities in Severe Pneumonia. Am. J. Respir. Crit. Care Med..

[219] Schultz M.J., Millo J., Levi M., Hack C.E., Weverling G.J., Garrard C.S., van der Poll T. (2004). Local activation of coagulation and inhibition of fibrinolysis in the lung during ventilator associated pneumonia. Thorax.

[220] Koyama K., Katayama S., Tonai K., Shima J., Koinuma T., Nunomiya S. (2019). Biomarker profiles of coagulopathy and alveolar epithelial injury in acute respiratory distress syndrome with idiopathic/immune-related disease or common direct risk factors. Crit. Care.

[221] Miyoshi S., Ito R., Katayama H., Dote K., Aibiki M., Hamada H., Okura T., Higaki J. (2014). Combination therapy with sivelestat and recombinant human soluble thrombomodulin for ARDS and DIC patients. Drug Des. Dev. Ther..

[222] Takahashi Y., Matsutani N., Dejima H., Nakayama T., Okamura R., Uehara H., Kawamura M. (2016). Therapeutic potential of recombinant thrombomodulin for lung injury after pneumonectomy via inhibition of high-mobility group box 1 in mice. J. Trauma Acute Care Surg..

[223] Kudo D., Toyama M., Aoyagi T., Akahori Y., Yamamoto H., Ishii K., Kanno E., Maruyama R., Kaku M., Kushimoto S. (2013). Involvement of high mobility group box 1 and the therapeutic effect of recombinant thrombomodulin in a mouse model of severe acute respiratory distress syndrome. Clin. Exp. Immunol..

[224] Juschten J., Ingelse S.A., Maas M.A.W., Girbes A.R.J., Juffermans N.P., Schultz M.J., Tuinman P.R. (2019). Antithrombin plus alpha-1 protease inhibitor does not affect coagulation and inflammation in two murine models of acute lung injury. Intensive Care Med. Exp..

[225] Ferrer-Acosta Y., Gonzalez M., Fernandez M., Valance W.A. (2014). Emerging Roles for Platelets in Inflammation and Disease. J. Infect. Dis. Ther..

[226] Gale N.W., Yancopoulos G.D. (1999). Growth factors acting via endothelial cell-specific receptor tyrosine kinases: VEGFs, Angiopoietins, and ephrins in vascular development. Genes Dev..

[227] Plouët J., Schilling J., Gospodarowicz D. (1989). Isolation and characterization of a newly identified endothelial cell mitogen produced by AtT-20 cells. EMBO J..

[228] Senger D.R., Galli S.J., Dvorak A.M., Perruzzi C.A., Harvey V.S., Dvorak H.F. (1983). Tumor cells secrete a vascular permeability factor that promotes accumulation of ascites fluid. Science.

[229] Medford A.R.L. (2006). Vascular endothelial growth factor (VEGF) in acute lung injury (ALI) and acute respiratory distress syndrome (ARDS): Paradox or paradigm?. Thorax.

[230] Maitre B., Boussat S., Jean D., Gouge M., Brochard L., Housset B., Adnot S., Delclaux C. (2001). Vascular endothelial growth factor synthesis in the acute phase of experimental and clinical lung injury. Eur. Respir. J..

[231] Abadie Y., Bregeon F., Papazian L., Lange F., Chailley-Heu B., Thomas P.A., Duvaldestin P., Adnot S., Maitre B., Delclaux C. (2005). Decreased VEGF concentration in lung tissue and vascular injury during ARDS. Eur. Respir. J..

[232] Azamfirei L., Gurzu S., Solomon R., Copotoiu R., Copotoiu S., Jung I., Tilinca M., Branzaniuc K., Corneci D., Szederjesi J. (2010). Vascular endothelial growth factor: A possible mediator of endothelial activation in acute respiratory distress syndrome. Minerva Anestesiol..

[233] Medford A.R., Ibrahim N.B., Millar A.B. (2009). Vascular endothelial growth factor receptor and coreceptor expression in human acute respiratory distress syndrome. J. Crit. Care.

[234] Perkins G., Roberts J., McAuley D., Armstrong L., Millar A., Gao F., Thickett D.R. (2005). Regulation of vascular endothelial growth factor bioactivity in patients with acute lung injury. Thorax.

[235] Thickett D.R., Armstrong L., Christie S.J., Millar A.B. (2001). Vascular Endothelial Growth Factor May Contribute to Increased Vascular Permeability in Acute Respiratory Distress Syndrome. Am. J. Respir. Crit. Care Med..

[236] Gaudry M., Bregerie O., Andrieu V., El Benna J., Pocidalo M.A., Hakim J. (1997). Intracellular Pool of Vascular Endothelial Growth Factor in Human Neutrophils. Blood.

[237] Thickett D.R., Armstrong L., Millar A.B. (2002). A Role for Vascular Endothelial Growth Factor in Acute and Resolving Lung Injury. Am. J. Respir. Crit. Care Med..

[238] Varet J., Douglas S.K., Gilmartin L., Medford A.R.L., Bates D.O., Harper S.J., Millar A.B. (2010). VEGF in the lung: A role for novel isoforms. Am. J. Physiol. Cell. Mol. Physiol..

[239] Medford A.R.L. (2005). Vascular endothelial growth factor gene polymorphism and acute respiratory distress syndrome. Thorax.

[240] Zhai R., Gong M.N., Zhou W., Thompson T.B., Kraft P., Su L., Christiani D.C. (2007). Genotypes and haplotypes of the VEGF gene are associated with higher mortality and lower VEGF plasma levels in patients with ARDS. Thorax.

[241] Yang S., Cao S., Li J., Chang J. (2011). Association between Vascular Endothelial Growth Factor + 936 Genotype and Acute Respiratory Distress Syndrome in a Chinese Population. Genet. Test. Mol. Biomark..

[242] Medford A.R.L., Douglas S.K., Godinho S.I., Uppington K.M., Armstrong L., Gillespie K.M., Van Zyl B., Tetley T.D., Ibrahim N., Millar A.B. (2009). Vascular Endothelial Growth Factor (VEGF) isoform expression and activity in human and murine lung injury. Respir. Res..

[243] Lin C.-K., Lin Y.-H., Huang T.-C., Shi C.-S., Yang C.-T., Yang Y.-L. (2019). VEGF mediates fat embolism-induced acute lung injury via VEGF receptor 2 and the MAPK cascade. Sci. Rep..

[244] Kaner R.J., Ladetto J.V., Singh R., Fukuda N., Matthay M.A., Crystal R.G. (2000). Lung Overexpression of the Vascular Endothelial Growth Factor Gene Induces Pulmonary Edema. Am. J. Respir. Cell Mol. Biol..

[245] Godzich M., Hodnett M., Frank J.A., Su G., Pespeni M., Angel A., Howard M.B., Matthay M.A., Pittet J.-F. (2006). Activation of the stress protein response prevents the development of pulmonary edema by inhibiting VEGF cell signaling in a model of lung ischemia-reperfusion injury in rats. FASEB J..

[246] Watanabe M., Boyer J.L., Crystal R.G. (2009). Genetic Delivery of Bevacizumab to Suppress Vascular Endothelial Growth Factor-Induced High-Permeability Pulmonary Edema. Hum. Gene Ther..

[247] Compernolle V., Brusselmans K., Acker T., Hoet P., Tjwa M., Beck H., Plaisance S., Dor Y., Keshet E., Lupu F. (2002). Loss of HIF-2α and inhibition of VEGF impair fetal lung maturation, whereas treatment with VEGF prevents fatal respiratory distress in premature mice. Nat. Med..

[248] Maisonpierre P.C., Suri C., Jones P.F., Bartunkova S., Wiegand S.J., Radziejewski C., Compton D., McClain J., Aldrich T.H., Papadopoulos N. (1997). Angiopoietin-2, a Natural Antagonist for Tie2 That Disrupts in vivo Angiogenesis. Science.

[249] Thurston G., Rudge J.S., Ioffe E., Zhou H., Ross L., Croll S.D., Glazer N., Holash J., McDonald D.M., Yancopoulos G.D. (2000). Angiopoietin-1 protects the adult vasculature against plasma leakage. Nat. Med..

[250] Fiedler U., Reiss Y., Scharpfenecker M., Grunow V., Koidl S., Thurston G., Gale N.W., Witzenrath M., Rosseau S., Suttorp N. (2006). Angiopoietin-2 sensitizes endothelial cells to TNF-α and has a crucial role in the induction of inflammation. Nat. Med..

[251] Roviezzo F., Tsigkos S., Kotanidou A., Bucci M., Brancaleone V., Cirino G., Papapetropoulos A. (2005). Angiopoietin-2 Causes Inflammation in Vivo by Promoting Vascular Leakage. J. Pharmacol. Exp. Ther..

[252] Parikh S.M., Mammoto T., Schultz A., Yuan H.-T., Christiani D.C., Karumanchi S.A., Sukhatme V.P. (2006). Excess Circulating Angiopoietin-2 May Contribute to Pulmonary Vascular Leak in Sepsis in Humans. PLoS Med..

[253] Gallagher D.C., Parikh S.M., Balonov K., Miller A., Gautam S., Talmor D., Sukhatme V.P. (2008). Circulating angiopoietin 2 correlates with mortality in a surgical population with acute lung injury/adult respiratory distress syndrome. Shock.

[254] Van Der Heijden M., Amerongen G.P.V.N., Koolwijk P., Van Hinsbergh V.W.M., Groeneveld A.B.J. (2008). Angiopoietin-2, permeability oedema, occurrence and severity of ALI/ARDS in septic and non-septic critically ill patients. Thorax.

[255] Ando M., Miyazaki E., Abe T., Ehara C., Goto A., Masuda T., Nishio S., Fujisaki H., Yamasue M., Ishii T. (2016). Angiopoietin-2 expression in patients with an acute exacerbation of idiopathic interstitial pneumonias. Respir. Med..

[256] Zhong M., Zhang L., Wang F., Peng S., Zhang J., Xuan G. (2014). The levels of angiopoietin-2 in patients with acute respiratory distress syndrome and its value on prognosis. Zhonghua Wei Zhong Bing Ji Jiu Yi Xue.

[257] Zinter M.S., Spicer A.C., Orwoll B., Alkhouli M., Dvorak C.C., Calfee C.S., Matthay M.A., Sapru A. (2016). Plasma angiopoietin-2 outperforms other markers of endothelial injury in prognosticating pediatric ARDS mortality. Am. J. Physiol. Cell. Mol. Physiol..

[258] Tsangaris I., Tsantes A., Vrigkou E., Kopterides P., Pelekanou A., Zerva K., Antonakos G., Konstantonis D., Mavrou I., Tsaknis G. (2017). Angiopoietin-2 Levels as Predictors of Outcome in Mechanically Ventilated Patients with Acute Respiratory Distress Syndrome. Dis. Markers.

[259] Calfee C.S., Gallagher D., Abbott J., Thompson B.T., Matthay M.A. (2012). Plasma angiopoietin-2 in clinical acute lung injury. Crit. Care Med..

[260] Ma S., Zhao M.-L., Wang K., Yue Y.-F., Sun R.-Q., Zhang R.-M., Wang S.-F., Sun G., Xie H.-Q., Yu Y. (2020). Association of Ang-2, vWF, and EVLWI with risk of mortality in sepsis patients with concomitant ARDS: A retrospective study. J. Formos. Med. Assoc..

[261] Reilly J.P., Wang F., Jones T.K., Palakshappa J.A., Anderson B.J., Shashaty M.G.S., Dunn T.G., Johansson E.D., Riley T.R., Lim B. (2018). Plasma angiopoietin-2 as a potential causal marker in sepsis-associated ARDS development: Evidence from Mendelian randomization and mediation analysis. Intensive Care Med..

[262] Araujo C.B., de Oliveira Neves F.M., de Freitas D.F., Arruda B.F.T., de Macedo Filho L.J.M., Salles V.B., Meneses G.C., Martins A.M.C., Liborio A.B. (2019). Angiopoietin-2 as a predictor of acute kidney injury in critically ill patients and association with ARDS. Respirology.

[263] Li F., Yin R., Guo Q. (2020). Circulating angiopoietin-2 and the risk of mortality in patients with acute respiratory distress syndrome: A systematic review and meta-analysis of 10 prospective cohort studies. Ther. Adv. Respir. Dis..

[264] Su L., Zhai R., Sheu C.-C., Gallagher D.C., Gong M.N., Tejera P., Thompson B.T., Christiani D.C. (2009). Genetic variants in the angiopoietin-2 gene are associated with increased risk of ARDS. Intensive Care Med..

[265] Wada T., Jesmin S., Gando S., Yanagida Y., Mizugaki A., Sultana S.N., Zaedi S., Yokota H. (2013). The role of angiogenic factors and their soluble receptors in acute lung injury (ALI)/acute respiratory distress syndrome (ARDS) associated with critical illness. J. Inflamm..

[266] Ware L.B., Zhao Z., Koyama T., Brown R.M., Semler M.W., Janz D.R., May A.K., Fremont R.D., Matthay M.A., Cohen M.J. (2017). Derivation and validation of a two-biomarker panel for diagnosis of ARDS in patients with severe traumatic injuries. Trauma Surg. Acute Care Open.

[267] Xu W., Song Y. (2017). Biomarkers for patients with trauma associated acute respiratory distress syndrome. Mil. Med. Res..

[268] Kümpers P., Gueler F., David S., Van Slyke P., Dumont D.J., Park J.-K., Bockmeyer C.L., Parikh S.M., Pavenstädt H., Haller H. (2011). The synthetic Tie2 agonist peptide vasculotide protects against vascular leakage and reduces mortality in murine abdominal sepsis. Crit. Care.

[269] David S., Ghosh C.C., Kümpers P., Shushakova N., Van Slyke P., Khankin E.V., Karumanchi S.A., Dumont D., Parikh S.M. (2011). Effects of a synthetic PEG-ylated Tie-2 agonist peptide on endotoxemic lung injury and mortality. Am. J. Physiol. Cell. Mol. Physiol..

[270] Lomas-Neira J.L., Heffernan D.S., Ayala A., Monaghan S.F. (2016). Blockade of Endothelial Growth Factor, Angiopoietin-2, Reduces Indices of Ards and Mortality in Mice Resulting from the Dual-Insults of Hemorrhagic Shock and Sepsis. Shock.

[271] Stiehl T., Thamm K., Kaufmann J., Schaeper U., Kirsch T., Haller H., Santel A., Ghosh C.C., Parikh S.M., David S. (2014). Lung-Targeted RNA Interference Against Angiopoietin-2 Ameliorates Multiple Organ Dysfunction and Death in Sepsis. Crit. Care Med..

[272] van der Heijden M., van Nieuw Amerongen G.P., Chedamni S., van Hinsbergh V.W., Johan Groeneveld A.B. (2009). The angiopoietin-Tie2 system as a therapeutic target in sepsis and acute lung injury. Expert Opin. Ther. Targets.

[273] Zhu N., Zhang D., Wang W., Li X., Yang B., Song J., Zhao X., Huang B., Shi W., Lu R. (2020). A Novel Coronavirus from Patients with Pneumonia in China, 2019. N. Engl. J. Med..

[274] Amraei R., Rahimi N. (2020). COVID19, Renin-Angiotensin System and Endothelial Dysfunction. Cells.

[275] Kumar A., Narayan R.K., Kumari C., Faiq M.A., Kulandhasamy M., Kant K., Pareek V. (2020). SARS-CoV-2 cell entry receptor ACE2 mediated endothelial dysfunction leads to vascular thrombosis in COVID-19 patients. Med. Hypotheses.

[276] Ackermann M., Verleden S.E., Kuehnel M., Haverich A., Welte T., Laenger F., Vanstapel A., Werlein C., Stark H., Tzankov A. (2020). Pulmonary Vascular Endothelialitis, Thrombosis, and Angiogenesis in Covid-19. N. Engl. J. Med..

[277] Smadja D.M., Guerin C.L., Chocron R., Yatim N., Boussier J., Gendron N., Khider L., Hadjadj J., Goudot G., DeBuc B. (2020). Angiopoietin-2 as a marker of endothelial activation is a good predictor factor for intensive care unit admission of COVID-19 patients. Angiogenesis.

[278] Gustafson D., Raju S., Wu R., Ching C., Veitch S., Rathnakumar K., Boudreau E., Howe K.L., Fish J.E. (2020). Overcoming Barriers: The Endothelium as a Linchpin of Coronavirus Disease 2019 Pathogenesis?. Arterioscler. Thromb. Vasc. Biol..

[279] Giamarellos-Bourboulis E.J., Netea M.G., Rovina N., Akinosoglou K., Antoniadou A., Antonakos N., Damoraki G., Gkavogianni T., Adami M.-E., Katsaounou P. (2020). Complex Immune Dysregulation in COVID-19 Patients with Severe Respiratory Failure. Cell Host Microbe.

[280] Ciceri F., Beretta L., Scandroglio A.M., Colombo S., Landoni G., Ruggeri A., Peccatori J., D’Angelo A., De Cobelli F., Rovere-Querini P. (2020). Microvascular COVID-19 lung vessels obstructive thromboinflammatory syndrome (MicroCLOTS): An atypical acute respiratory distress syndrome working hypothesis. Crit. Care Resusc..

[281] Goshua G., Pine A.B., Meizlish M.L., Chang C.-H., Zhang H., Bahel P., Baluha A., Bar N., Bona R.D., Burns A.J. (2020). Endotheliopathy in COVID-19-associated coagulopathy: Evidence from a single-centre, cross-sectional study. Lancet Haematol..

[282] Frank J.A., McAuley D.F., Gutierrez J.A., Daniel B.M., Dobbs L., Matthay M.A. (2005). Differential effects of sustained inflation recruitment maneuvers on alveolar epithelial and lung endothelial injury. Crit. Care Med..

